# In Situ GISAXS Study of IZO Deposition via Magnetron Sputtering for Optoelectronic Devices: Film Growth and Ion Bombardment‐Induced Degradation Dynamics

**DOI:** 10.1002/advs.202516853

**Published:** 2025-11-07

**Authors:** Huaying Zhong, Marlene Sophie Härtel, Wei Chen, Lukas V. Spanier, Shanshan Yin, Jiahuan Zhang, Bertwin Bilgrim Otto Seibertz, Bernd Szyszka, Steve Albrecht, Matthias Schwartzkopf, Stephan V. Roth, Peter Müller‐Buschbaum

**Affiliations:** ^1^ Technical University of Munich TUM School of Natural Sciences Department of Physics Chair for Functional Materials James‐Franck‐Str. 1 85748 Garching Germany; ^2^ Helmholtz‐Zentrum Berlin für Materialien und Energie Hahn‐Meitner‐Platz 1 14109 Berlin Germany; ^3^ College of Engineering Physics and Center for Intense Laser Application Technology Shenzhen Technology University Shenzhen 518118 China; ^4^ School of Mathematics and Physics and Jiangsu Key Laboratory of Clean Energy Storage and Conversion Jiangsu University of Technology Changzhou 213001 China; ^5^ Technische Universität Berlin Straße des 17 10623 Berlin Germany; ^6^ Deutsches Elektronen‐Synchrotron DESY Notkestraße 85 22607 Hamburg Germany; ^7^ KTH Royal Institute of Technology Department of Fibre and Polymer Technology Teknikringen 56‐58 Stockholm 11428 Sweden

**Keywords:** degradation dynamics, growth dynamics, ion bombardment, IZO, sputter deposition

## Abstract

Magnetron sputtering is a well‐established fabrication technique in industry for the deposition of transparent conductive oxides toward optoelectronic device applications. However, the bombardment with highly energetic O^−^ ions can damage underlying sensitive layers of devices or the growing film itself, being a critical issue. Its substrate‐dependent impact is not fully understood. Herein, the early‐stage dynamics of film growth and ion‐bombardment‐induced degradation are studied independently by applying two distinct templates as substrates for indium zinc oxide (IZO) deposition via radio frequency magnetron sputtering, with real‐time monitoring via in situ grazing‐incidence small‐angle X‐ray scattering (GISAXS). X‐ray reflectivity results reveal that O^−^ ion‐bombardment results in a reduced density and modified surface morphology of spin‐coated ZnO nanoparticle (NP) films, despite a relatively high working pressure, whereas commercially sputter‐coated polycrystalline indium tin oxide (ITO) films exhibit stronger resistance, enabling the successful formation of an IZO layer. Quantitative analysis of GISAXS data shows that the growth regimes of IZO deposited on the ITO film undergo the stages of nucleation, adsorption‐driven coalescence, and layer formation. Conversely, the degradation dynamics on the ZnO NP film exhibit a cyclical pattern under ion bombardment, characterized by alternating phases of adsorption‐desorption equilibrium, physical degradation, reestablished adsorption‐desorption equilibrium, and surface amorphization.

## Graphical abstract

The dynamics of film growth and ion bombardment‐induced degradation during the film deposition via radio frequency magnetron sputtering of indium zinc oxide are probed with real‐time monitoring via in situ grazing‐incidence small‐angle X‐ray scattering, causing physical degradation on a spin‐coated ZnO NP film and film growth on a robust, commercially sputtered indium tin oxide film.

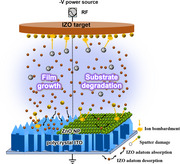

## 1 Introduction

Transparent conductive oxides (TCOs), featuring high electrical conductivity and low optical absorption from the ultraviolet (UV) to the infrared (IR) regime due to their large optical bandgap, have been widely applied in optoelectronic devices, such as photovoltaics,^[^ ^1^ ^]^ thin film transistors,^[^ ^2^ ^]^ light‐emitting diodes (LEDs),^[^ ^3^ ^]^ and photodetectors.^[^ ^4^ ^]^ TCOs cover many compounds, including In_2_O_3_‐based films, SnO_2_‐based films, ZnO‐based films, and other multi‐compound materials.^[^ ^5^ ^]^ In addition to indium tin oxide (ITO) films, which are perhaps the most widely used transparent front electrodes in optoelectronic devices, ZnO nanoparticle (NP) films have been commonly used as an electron transport layer (ETL) owing to their low costs, non‐toxicity, and the abundance of chemical elements like Zn and O in nature. Particularly, ITO/ZnO stack has been utilized for electron transport and extraction in many optoelectronic devices, such as PbS quantum dot (QD) solar cells, due to its electron affinity alignment with 1.3 eV bandgap PbS QD films and high electron mobility.^[^ ^6^ ^]^ Besides crystalline TCOs, amorphous TCOs like indium zinc oxide (IZO) are particularly attractive in various optoelectronic applications, with respect to their high transmittance in the visible and near‐infrared range in combination with a low sheet resistance.^[^ ^7^ ^]^ Moreover, IZO is sometimes applied as a buffering layer or ETL for bandgap alignment to improve the overall device performance in various types of solar cells, such as inverted polymer solar cells,^[^ ^8^ ^]^ perovskite solar cells,^[^ ^9^ ^]^ and PbS QD solar cells.^[^ ^1^ ^]^

Among the various physical vapor deposition (PVD) techniques for TCO fabrication, magnetron sputtering is the most widespread technique for both laboratory‐scale research and commercial applications, owing to the high reproducibility and excellent scalability of dense and homogeneous TCO films.^[^ ^10^, ^11^ ^]^ In magnetron sputtering, the sputtering deposition process involves a wide range of species, such as the sputtered atoms from the target surface, positive and negative ions formed in plasma, reflected atoms and neutralized ions from the target surface, and negative ions formed at the target surface.^[^ ^11^ ^]^ Notably, the kinetic energy of negative O^−^ ions generated at the target surface, which is closely linked to the magnitude of discharge voltage, can reach up to several hundreds of eV, making it the highest among plasma species and photons in the sputtering process.^[^ ^10^, ^12^ ^]^ Therefore, the unwanted damage to the sensitive underlying layers or the growing film itself during the film deposition, commonly caused by the ion bombardment of negative ions, has been a critical issue.^[^ ^13^, ^14^ ^]^ In magnetron sputtering, the collision mean free path λ, corresponding to the average distance a particle can travel between two subsequent collisions with another particle, is an important measure for deposition characteristics. By using an energy‐dependent scattering collision cross section δ(*E*) introduced by Robinson,^[^ ^15^ ^]^ the collision mean free path λ is proportional to *T*/*P*δ(*E*), with gas temperature (T) and working pressure P.^[^ ^16^ ^]^ A shorter mean free path leads to more collisions between sputtered particles and gas atoms, leading to thermalization to reduce their kinetic energy. Additionally, the average number of collisions a sputtered particle experiences on its way from the target to the substrate (d) is defined as *N* = *d*/λ.^[^ ^17^ ^]^ Therefore, adjusting sputtering parameters, which include increasing working pressure P, lowering the power density on the target, and extending the target‐to‐substrate distance *d*, are established strategies to reduce bombardment‐induced damage to the sensitive substrates or films. In optoelectronic devices, the growing TCO films, the underlying charge transport layers (CTL), and the active materials can be susceptible to particle bombardment during the TCO deposition. Previous studies primarily focused on the effect of sputter‐induced damage on device performance.^[^ ^10^, ^13^ ^]^ However, the mechanism dynamics of O^−^ ion bombardment and its interaction with the substrate during the sputtering process still remain unclear.

In this study, the focus is on understanding both the film growth dynamics and the ion bombardment‐induced degradation process during the TCO film deposition process. The early stages of IZO sputter deposition on different templates via radio frequency (RF) sputtering are monitored in real‐time using in situ GISAXS measurements. Two distinct templates, the commercially sputtered ITO layer (glass/ITO, denoted Template T1) and the spin‐coated ZnO NP film on ITO (glass/ITO/ZnO, denoted Template T2), are individually used as substrates for IZO sputter deposition at room temperature (RT). Notably, the set sputtering conditions, involving a relatively high working pressure of 3.5 × 10^−2^ mbar in pure Ar gas and a low deposition rate of 10 nm/h, were chosen to test the theory, whether a high working gas pressure can effectively reduce the kinetic energy of damaging sputter particles, and to resolve the dynamics of the sputtered particles and substrate interaction. A successful IZO film formation on ITO film on Template T1 and a decreased density of ZnO NP film on Template T2 with unchanged thickness, as confirmed by X‐ray reflectivity (XRR) results, demonstrate that the spin‐coated ZnO NP film is more sensitive to the ion bombardment‐induced damage than the commercially sputter‐coated ITO film despite the high working pressure. Scanning electron microscopy (SEM) was performed to investigate the effects of IZO sputtering on morphology changes of each template. According to the modeling of in situ GISAXS data, the layer growth mode of IZO deposition on the ITO film of the Template T1 is identified based on the different growth regimes, and the dynamics of O^−^ ion bombardment‐induced physical degradation on ZnO surface of the Template T2 is also quantitatively analyzed. Altogether, we visualize the growth of IZO film formation and the O^−^ ion bombardment‐induced degradation process during the IZO sputtering. This study sheds light on a fundamental understanding of film growth and ion bombardment‐induced degradation mechanisms during TCO sputtering and demonstrates that reducing sputtering damage is a complex task and not yet well understood. Furthermore, sputtering parameters and the kinetic energy of ions can significantly influence the resulting film properties.

## 2 Result and Discussion

### 2.1 Sputter Conditions

**Figure 1:**
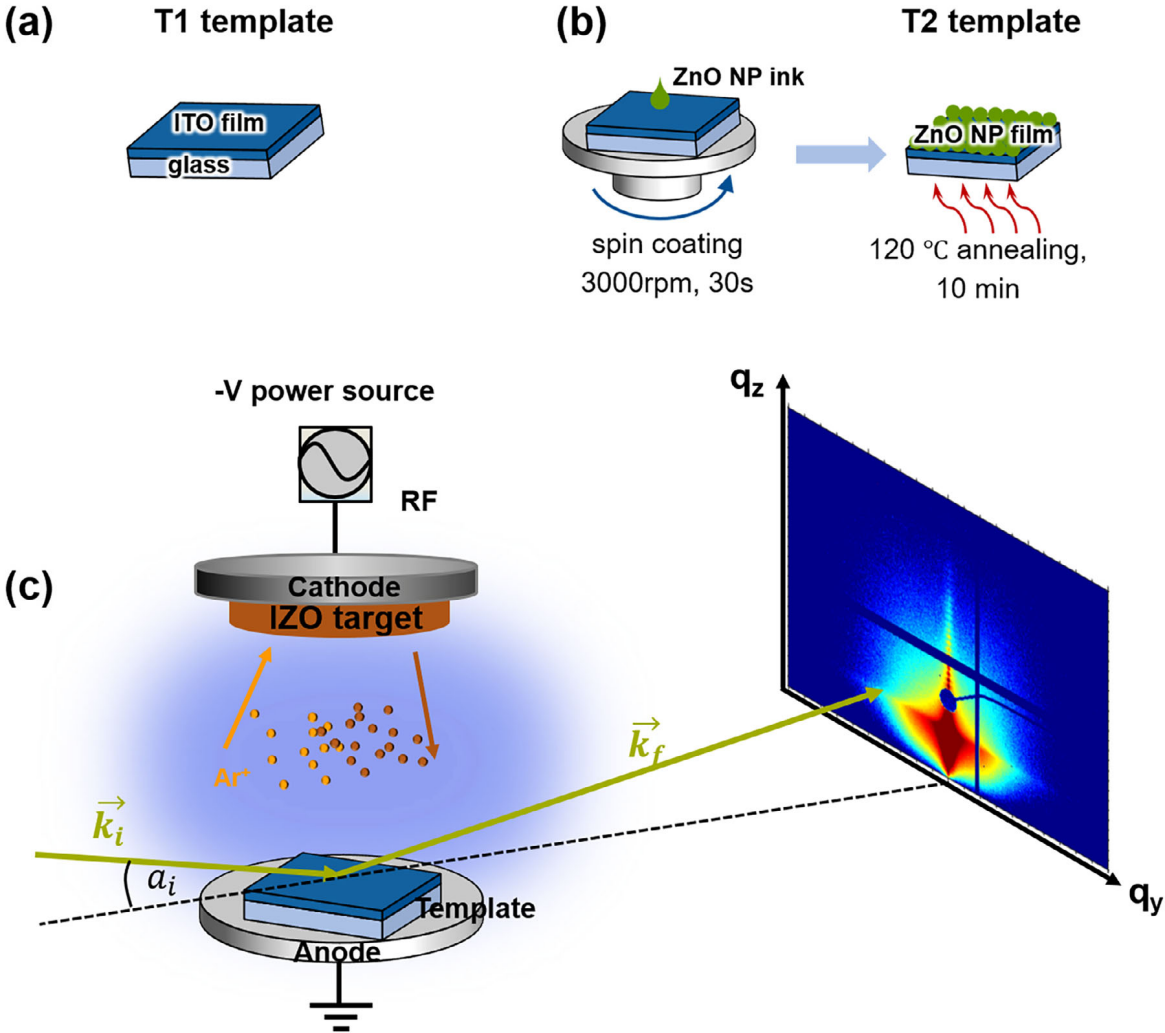
a) Sketch of the Template T1 and b) the fabrication process of the Template T2. c) Schematic illustration of in situ GISAXS setup for the sputter deposition of IZO on a template. Argon plasma is used to sputter IZO. The argon ions and sputtered IZO atoms are indicated as golden spheres and orange spheres, respectively. The incident X‐ray beam and an exemplary scattered beam are shown in dark green, and the scattered X‐ray signal is probed on a 2D detector.

The two templates used for the IZO sputter deposition are denoted with T1 and T2, respectively. The layer thickness for each template is measured by cross‐sectional SEM, as shown in Figure S1 (Supporting Information). The Template T1 comprises glass/ITO, while the Template T2 consists of glass/ITO/ZnO. The Template T1 (**Figure** **1**a) is a commercially sputtered ITO film on float glass with a thickness of 180 ± 20 nm, and the typical composition of the ITO film is a 90:10 wt.% ratio of In_2_O_3_ to SnO_2_. As illustrated in Figure 1b, the Template T2 is prepared by a spin‐coated ZnO NP thin film onto the above‐mentioned Template T1, and the thickness of the ZnO NP film is 25 ± 5 nm. An IZO target, with a composition of 90:10 wt.% ratio of In_2_O_3_ to ZnO, was applied for RF sputtering. As is sketched in Figure 1c, both the cleaned Template T1 and the prepared Template T2 are individually placed on the substrate holder in the RF sputter chamber for the in situ experiments, where IZO sputtering and GISAXS measurements are simultaneously performed. Further details are provided in the Experimental section.

The in situ sputtering experiments are conducted for 75 min at a low nominal sputter rate of 10 nm h^−1^, resolving the early‐stage growth regimes of IZO deposition on the ITO film of the Template T1. Additionally, a gas pressure of 3.5 × 10^−2^ mbar with pure argon as working gas, which is higher than the typical sputtering pressure used for TCOs fabrication, was chosen, in order to investigate the theory of reduced sputtered particle kinetic energy at high working gas pressures through thermalizing collisions. All sputtering conditions and measurement parameters for both templates are consistently maintained.

### 2.2 Initial Templates and Final Films After IZO Sputtering

**Figure 2:**
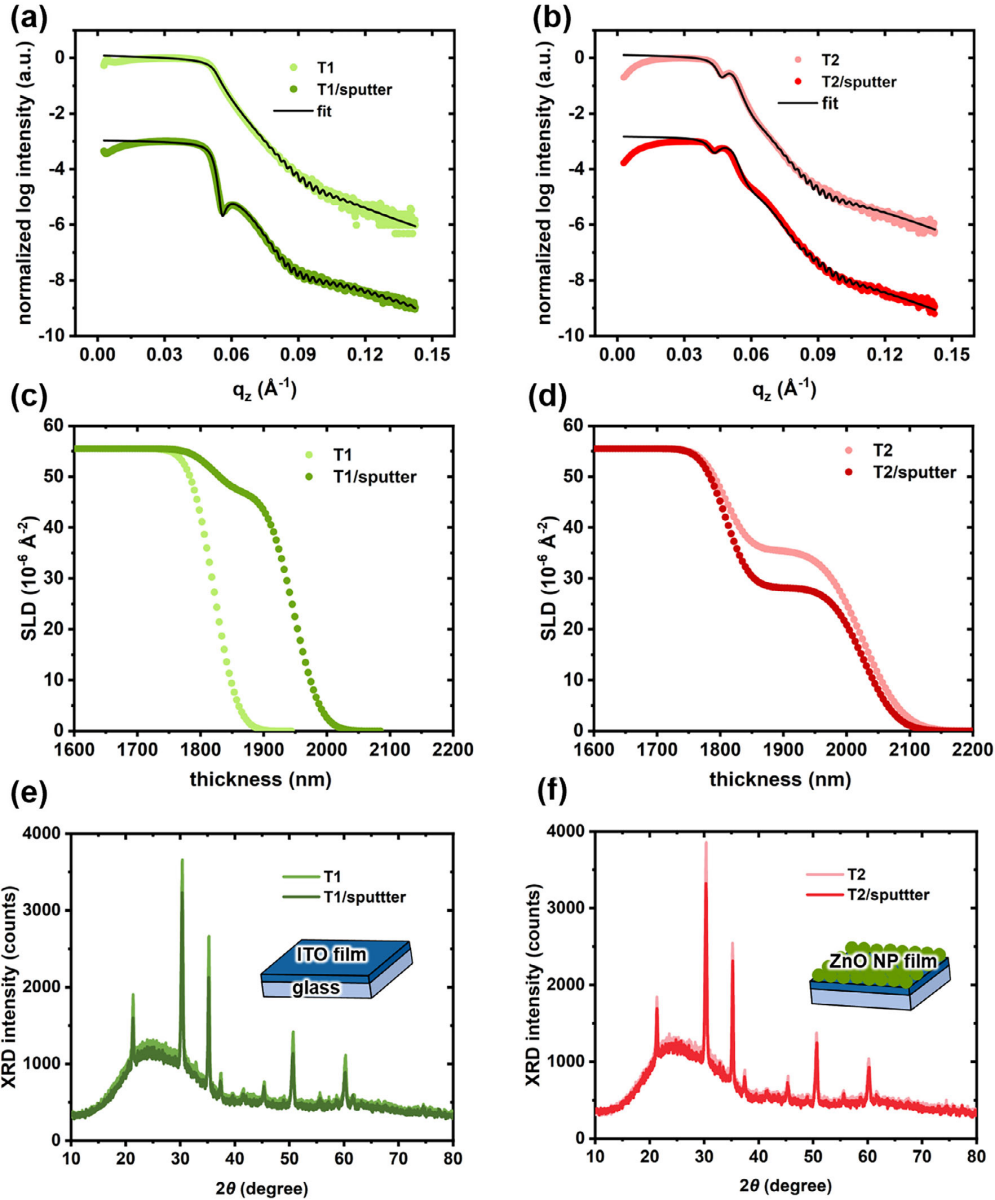
XRR data analysis reveals characteristic SLD profiles perpendicular to the film surface. XRR data (symbol) and fits (solid line) for the samples before and after IZO sputtering on a) the Template T1 and b) the Template T2. SLD profiles obtained from the fits are shown in c) the Template T1 and d) the Template T2. XRD data for the samples before and after IZO sputtering on e) the Template T1 and f) the Template T2.

To access the thickness, roughness, and scattering length density (SLD) of each layer (surface, bulk, and interface layer) in the multilayer samples, XRR is used. The XRR data are shown together with fits before and after IZO sputtering on the Template T1 and Template T2 in **Figure** **2**. The corresponding fit parameters are summarized in Tables S1 and S2 (Supporting Information). Due to over‐illumination of the samples by the X‐ray beam, the low *q_z_* range is excluded in the fits.^[^ ^18^ ^]^ All the XRR data exhibit pronounced Kiessig fringes (Figure 2a,b), which is attributed to the well‐defined ITO film, with a thickness of 176.6 ± 0.8 nm including an interfacial layer at the ITO/glass interface. Excluding the soda‐lime glass substrate and the infinite air layer on the top of each sample, a double‐layer model is applied for the Template T1 modeling, consisting of an ITO‐substrate interface and an ITO bulk layer. For the Template T1, the IZO particles are successfully sputtered on the ITO surface, forming an IZO film with a thickness of 12.6 ± 0.8 nm, including an interface layer of 1.6 ± 0.1 nm between the IZO layer and the ITO layer, as indicated by the pronounced bump in the XRR data of the T1/sputter sample in Figure 2a. The SLD profile is presented in Figure 2c. The SLD value of IZO (46.8 ± 0.1) × 10^−6^ Å^−2^ is lower than that of ITO (55.0 ± 0.3) × 10^−6^ Å^−2^, due to the lower mass density of the 10 wt.% ZnO in the IZO target compared with the 10 wt.% SnO_2_ in the ITO film. Moreover, the IZO deposition leads to an increase in surface roughness (as seen in Table S1, Supporting Information).

Based on the modeling results of the Template T1, a three‐layer model comprising the ITO glass substrate and a ZnO NP layer is applied to the Template T2. For the Template T2, the critical edges of the measured XRR curve at low and high *q_z_* positions correspond to ZnO and ITO (Figure 2b). The thickness of the ZnO surface layer is 21.6 ± 2 nm, consistent with 25 ± 5 nm in cross‐sectional SEM. As shown in Figure 2d, after 75 min of IZO sputtering on the Template T2, the SLD value of the surface layer reduces from (35.5 ± 0.5) × 10^−6^ Å^−2^ to (27.7 ± 0.3) × 10^−6^ Å^−2^, while the surface thickness remains relatively constant, accompanied by a reduction in surface roughness. Distinct changes in the XRR curves before and after the IZO sputtering on the templates can be observed in Figure S2b (Supporting Information). The counterintuitive phenomenon of a reduced surface roughness and SLD without a significant change in the thickness of the ZnO NP film can be attributed to surface amorphization of the crystalline ZnO NP film. It was reported that high‐energy ion sputtering could damage the crystalline structure of materials, leading to amorphization and consequently a smoother surface with fewer pronounced topographic features, especially at low temperatures. ^[^ ^19^, ^20^ ^]^

To verify if surface amorphization occurs during the IZO sputtering, we utilize X‐ray diffraction (XRD) to study the effect of ion bombardment on the structure of all measured samples. As shown in Figure S3 (Supporting Information), all samples exhibit diffraction peaks corresponding to In_2_O_3_ crystals with a body‐centered cubic crystal structure. This observation suggests that the strong In_2_O_3_ peaks from the ITO film dominate the diffraction patterns and mask the ZnO signal in the Template T2 and T2/sputter samples, owing to the substantial thickness difference between the ITO and ZnO layers. The broad peak observed at a scattering angle of 25° is ascribed to the glass substrate in all samples. To evaluate surface amorphization caused by ion bombardment for each Template, the XRD peak intensities of both templates before and after IZO sputtering are compared. As shown in Figure 2e, the diffraction peak intensity of the ITO crystal in the Template T1decreases after IZO deposition, even though the top IZO layer becomes slightly rougher according to XRR results (surface roughness rises from 2.94 to 3.30 nm), which would normally enhance the diffraction peak intensity. This reduction indicates that the ITO film undergoes surface amorphization induced by ion bombardment before the formation of the IZO film. Interestingly, for the Template T2 (Figure 2f), despite no direct ion sputtering exposed on the ITO film, the diffraction peak intensity corresponding to the ITO crystal also diminishes. The decrease can be attributed to reduced diffraction intensity of the smoothened ZnO surface due to its surface amorphization induced by the ion bombardment. This finding suggests that surface amorphization, as a form of physical degradation, occurs in both the ITO film of the Template T1 and the ZnO NP film of the Template T2 during the IZO sputtering, driven by the effect of high‐energy ion bombardment.

**Figure 3:**
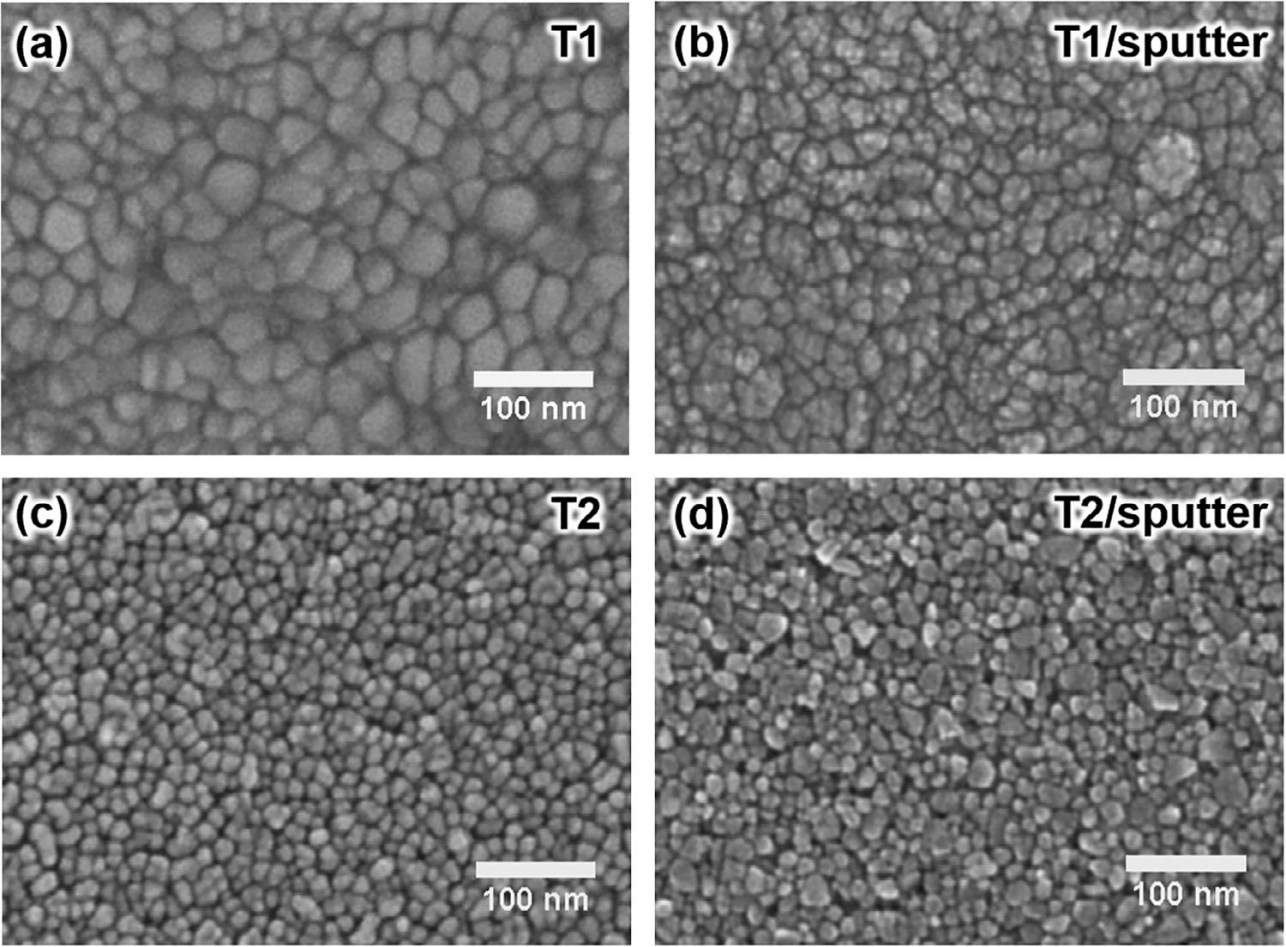
SEM images of the Template T1: a) before and b) after 75 min of IZO sputtering, and of the Template T2: c) before and d) after 75 min of IZO sputtering.

The changes in surface morphology after the IZO sputtering for 75 min are captured by SEM. By comparing **Figure** **3**a,b, it is evident that for the Template T1, the surface morphology transitions from large, polygonal domains (ranging in size from 10 nm to 30 nm) of the ITO film to a more granular structure after 75 min of IZO sputter deposition, indicating that the small IZO granular domains agglomerate with distinct grain boundaries and locally link end‐to‐end on larger ITO domains. This film growth behavior of granular domains was unexpected. Normally, sputter‐deposited IZO films are smooth and amorphous.^[^ ^21^ ^]^ The granular film growth is likely attributed to insufficient energy transfer of IZO particles with moderate kinetic energy of a few eVs (<50 eV), due to the thermalization through collisions with gas atoms under a high working pressure.^[^ ^22^ ^]^ This highlights the importance of process window control and the complexity of sputter deposition processes. For the Template T2 (Figure 3c), the porous ZnO NP film can effectively cover the underlying ITO film, with visible aggregates of small ZnO NPs. After 75 min of IZO sputtering (Figure 3d), the surface particle structures become irregular and the changes of their spatial configurations are distinct, indicating that the energetic ion bombardment strongly modifies the surface morphology of ZnO NP film.

Unlike SEM, which generally reflects the local morphological information in real space, grazing‐incidence small‐angle X‐ray scattering (GISAXS) is a complementary reciprocal space analysis method, allowing us to obtain statistically significant morphological changes of thin films.^[^ ^23^ ^]^ To qualitatively compare the consistency of the SEM images with the 2D GISAXS data regarding the statistical changes in the surface morphology, the SEM images taken in a large field and the ex situ 2D GISAXS data are shown in Figures S4 and S5 (Supporting Information), respectively. By comparing Figure S5a,b (Supporting Information), it is observed that the formation of the IZO thin film on the Template T1 significantly alters both the shape of the 2D scattering patterns and the intensity distributions, indicating the changes in the overall inner film morphology and structural configuration of the T1/sputter sample relative to the original T1 sample. However, only minor changes in intensity distribution are observed on the 2D GISAXS data of the Template T2 and the T2/sputter sample. To quantitatively analyze the implications of changes in intensity distribution, the 2D GISAXS data are processed using vertical and horizontal cuts for 1D data analysis.

### 2.3 In Situ Study of Film Growth and Degradation During Sputtering

In situ GISAXS measurements are conducted to study the structural changes quantitatively. In these experiments, an incident angle above all materials' critical angles (total external reflection) is used to obtain statistically relevant information about the entire film in terms of structure factors and form factors.^[^ ^24^ ^]^ In the total external reflection region, the intensity distribution, including dynamical effects, can be interpreted in the Distorted Wave Born Approximation (DWBA) framework.^[^ ^25^ ^]^ To eliminate errors from film heterogeneity, the in situ GISAXS data are extracted from the same spot on the sample surface.

Information about structures perpendicular to the sample surface, including the degree of correlation between interfaces, can be derived from the *q_z_* component of the scattering vector through vertical line cuts of the 2D GISAXS data. The Yoneda peak along the *q_z_* direction appears when the exit angle of the scattered X‐ray beam equals the critical angle, due to signal enhancement from the Vineyard effect,^[^ ^26^ ^]^ enabling the determination of the electron density of a specific material penetrated by the X‐ray beam. More details can be found in the Supporting Information. The selected 2D GISAXS data for the Template T1 and Template T2, showing the evolution of intensity distribution on the 2D detector during IZO sputtering, are presented in Figures S6 and S7 (Supporting Information), respectively. Vertical line cuts are performed on the selected 2D GISAXS data at *q_y_* = 0 nm^−1^ (Δ*q_y_* = 0.009 nm^−1^) along the *q_z_* direction.

**Figure 4:**
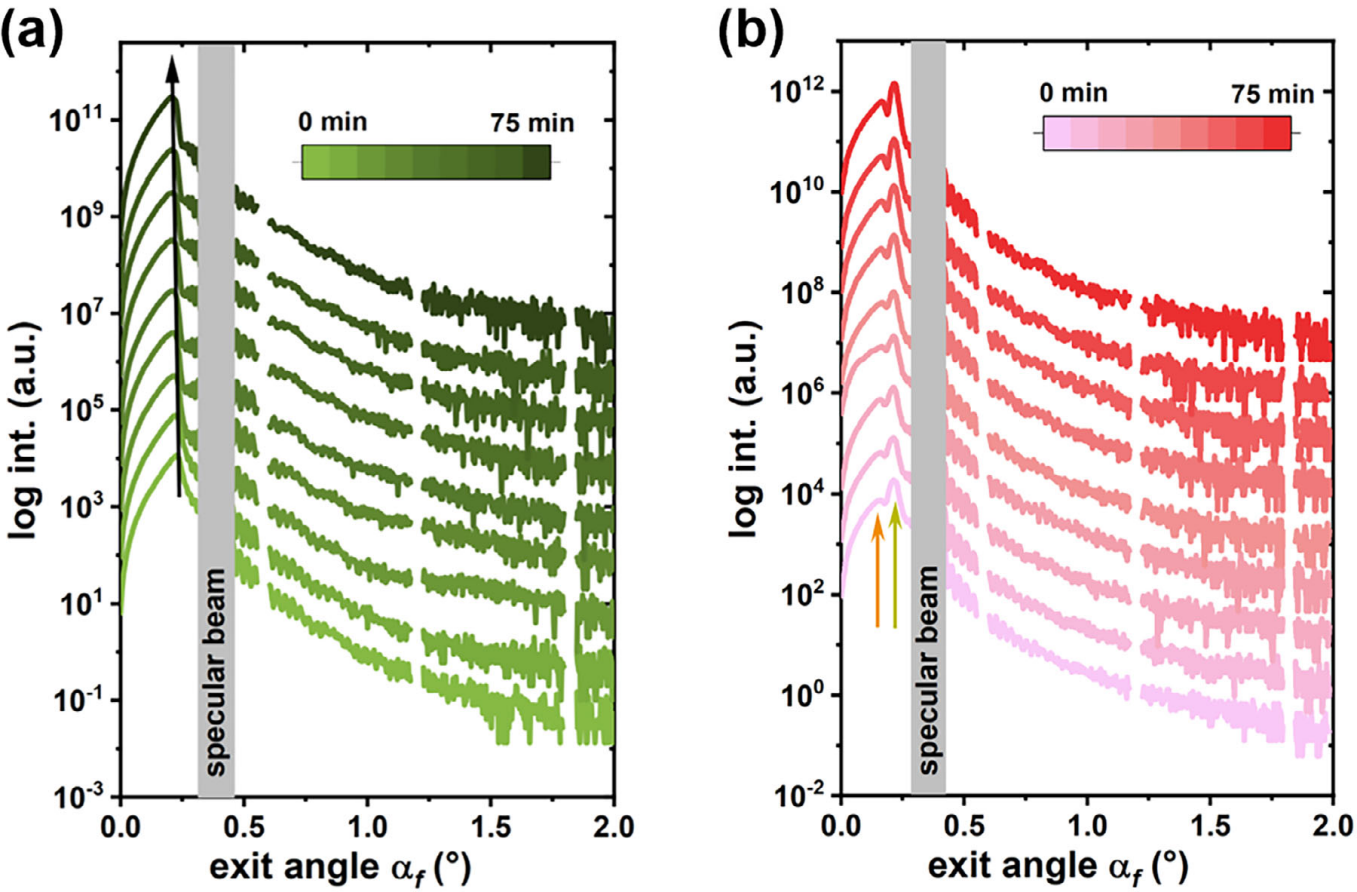
Representative vertical line cuts of the selected 2D GISAXS data at *q_y_* = 0 nm^−1^ (Δ*q_y_* = 0.009 nm^−1^ for a) the Template T1 and b) the Template T2. The black solid arrow guides the position shift of the Yoneda peak on the Template T1 over sputtering time. The orange and dark yellow arrows denote the Yoneda peak positions corresponding to the ZnO and ITO films.

Before IZO sputtering, the Template T1 exhibits a single, pronounced Yoneda peak from the ITO film at *q_z_* = 0.66 ± 0.01 nm^−1^ (*a_f_* = 0.23 ± 0.01°), as marked in Figure S6 (Supporting Information). The vertical line cut results, presented in **Figure** **4**a, reveal a gradual shift of the single Yoneda peak toward lower scattering angles as sputtering progresses. This trend aligns with the XRR findings, which show that an IZO film with a lower SLD is deposited on an ITO film with a higher SLD, suggesting that the growth of the IZO film predominantly influences the evolution of the Yoneda peak. For the Template T2, apart from the Yoneda peak from ITO film, another Yoneda peak attributed to ZnO NP film appears at *q_z_* = 0.58 ± 0.01 nm^−1^ (*a_f_* = 0.16 ± 0.01°), as marked in Figure S7 (Supporting Information). As presented in Figure 4b, the positions of both Yoneda peaks remain stable, while the shape of the Yoneda band (the region around the Yoneda peaks) below the critical angle of ZnO gradually evolves during sputtering. In comparison with the Template T1, this evolution indicates changes in the electron density distribution on the ZnO surface, which may be associated with interactions between energetic species in the plasma and the ZnO surface. This interpretation is supported by the XRR result, which reveals a decrease in surface density without a corresponding change in the thickness of the ZnO NP surface layer. In Figure S8 (Supporting Information), the variations in Yoneda peak positions on both templates over sputtering time are clearly visible.

Similar to the XRR curves, the correlated thickness obtained from the intensity fringes, caused by resonant diffuse scattering (RDS), ^[^ ^27^ ^]^ is also attributed to the ITO film. The RDS results of all samples are presented in Figure S9a (Supporting Information), and the intensity fringes in the scattering angle ranging from 0.44 °to 0.55 °are fitted using a Lorentzian function and multiple Gaussian functions, as presented in Figure S9b (Supporting Information). According to 1D Bragg‐condition *q_z_* = 2π/*d_corr_*, the vertically correlated interface distance *d_corr_* derived from RDS for both substrates remains at 190 ± 10 nm, corresponding to ITO film. This value aligns well with the XRR result and the nominal commercial specification. The reduced fringe amplitude observed in the T1/sputter sample compared with the Template T1 is attributed to a decrease in electron density contrast at the interface, converting from ITO‐air to ITO‐IZO after IZO deposition. In contrast, the fringe intensity remains unchanged for the Template T2 after the IZO sputtering, consistent with an unchanged electronic density contrast at the ITO‐ZnO interface.

To examine changes in lateral structures, such as the interference function, domain shape, size, and polydispersity, the horizontal line cut at the Yoneda peak of the surface material is performed, leveraging the enhanced scattering contribution from the material at this position.^[^ ^24^ ^]^ The selected horizontal line cuts are modeled within the framework of DWBA using the local monodisperse approximation (LMA), which relates to the effective interface approximation (EIA).^[^ ^28^ ^]^ The isotropic scattering intensity from the background in 2D GISAXS is modeled as a constant background. In this LMA‐EIA model approximation, all structural features are assumed to be arranged as lateral objects with defined size distributions. The error bars represent the fit tolerances of modeling larger than the distributions due to the assumption of orderly structures in the LMA.^[^ ^29^ ^]^ The growth evolution is depicted with the structure factors indicating the correlated inter‐domain distance *D*, form factors that define domain shape and domain size *R*, the size distribution σ, the distribution of correlated inter‐domain distance ω, aspect ratio of *2R/D*, and amplitude *I*.

**Figure 5:**
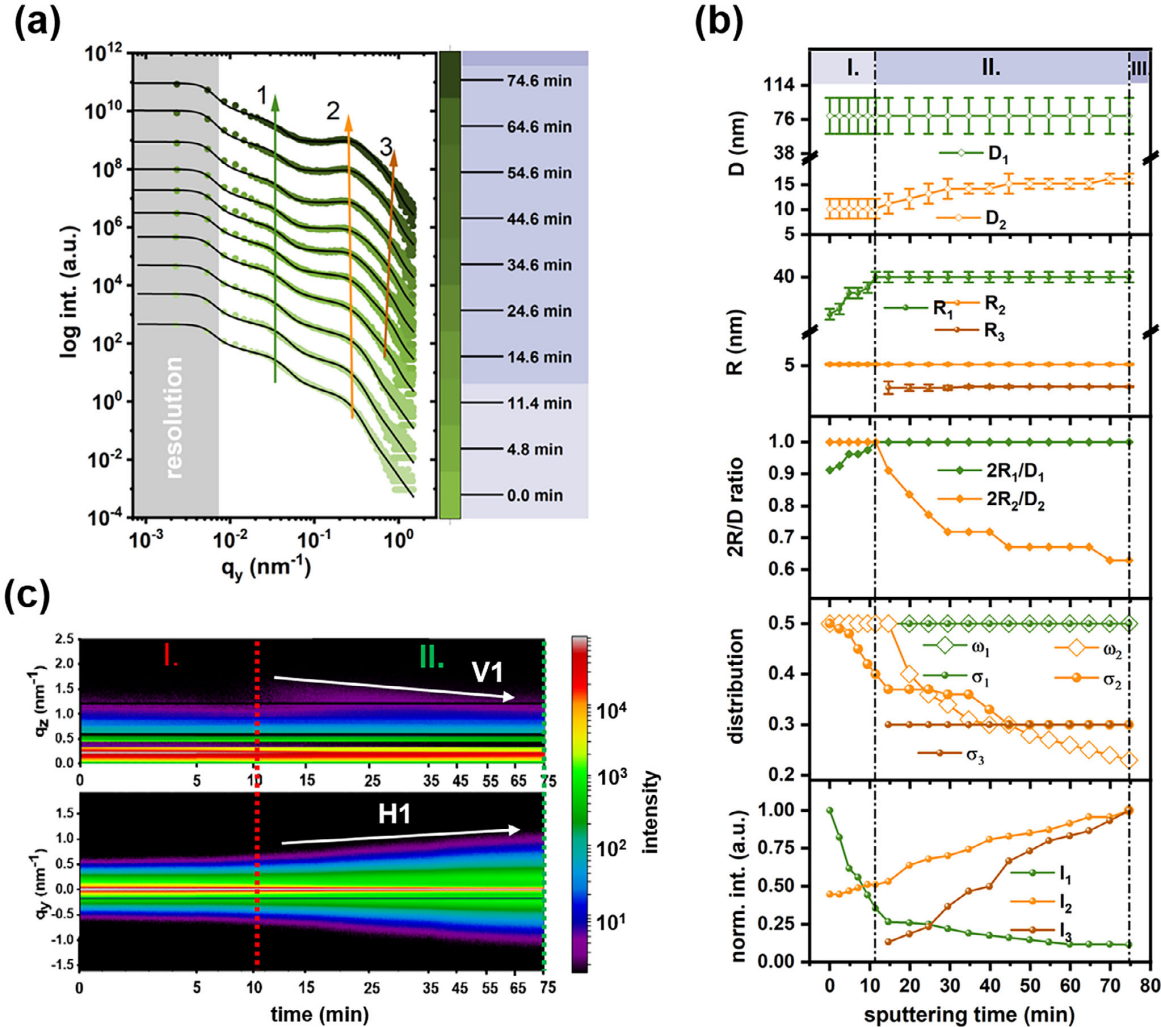
a) Selected horizontal line cuts from the 2D GISAXS data of the ITO‐templated film (Template T1) with increasing sputtering time. The fits are shown as black lines. Curves are shifted along the intensity axis for clarity of the presentation. The marked green, orange, and brown arrows show the features of structure 1, structure 2, and structure 3, respectively. b) Structure‐related parameters extracted from modeling data. The error bars represent the fit parameter tolerance. The three observed film growth regimes (I–III) are indicated. c) 2D mapping of all horizontal and vertical line cuts obtained from the 2D GISAXS data. The peak V1 represents the first maximum of the intensity modulation in the *q_z_* direction. The peak H1 represents the scattering intensity from structure 3 with reduced inter‐domain distance at the growth stage II.

To investigate the evolution of lateral structures of IZO growth on the Template T1, horizontal integrations of selected 2D GISAXS data are performed along the Yoneda peak region corresponding to the ITO film (*q_z_* = 0.648–0.660 nm^−1^). As presented in **Figure** **5**a, two primary features (denoted as structure 1 and structure 2) ascribed to the ITO film are observed for the Template T1 before sputtering, and the third feature, structure 3, emerges in the high *q_y_* range after 11.4 min of sputtering. Over time, predominant changes occur for each of these structures. The scattering feature of structure 1 becomes smeared out, while the scattering feature of structure 2 shifts to a lower *q_y_* position, indicating an increase in the correlated inter‐domain distance.^[^ ^30^ ^]^ Additionally, the more confined and distinct scattering feature of structure 2, with an increased peak‐to‐valley height, suggests a more orderly configuration of inner structures. In contrast, the scattering feature of structure 3 shifts toward higher *q_y_* values over sputter time. Prior to IZO sputtering, the ITO surface layer of the Template T1 is modeled into two structures identified with cylindrical form factors. As shown in Figure S10a (Supporting Information), s**tructure 1** comprises larger domains with an average radius *R_1_* of 36 nm, a size distribution σ_1_ of 50%, and a correlated inter‐domain distance *D_1_* of 80 nm with a distance distribution ω_1_ of 50%. **Structure 2**, representing smaller domains, has an average radius *R_2_* of 5.6 nm with a size distribution σ_2_ of 50%. Over the sputter time, it is evident that the large domain size increases with reduced amounts, while the number of small domains increases.

To understand the evolution of lateral structures, three distinct growth regimes of the IZO deposition on the Template T1 are illustrated, based on the quantitative modeling results of horizontal line cuts (Figure 5b). In growth regime I (0–11.4 min), the IZO deposition initiates, and morphological evolution is primarily governed by structure 1 and structure 2. The radius *R_1_* increases from 36 nm to 40 nm, accompanied by the aspect ratio *2R_1_/D_1_* slowly reaching up to 1, indicating the formation of a percolating layer that results in a decrease of the scattering amplitude *I_1_* due to reduced electron density contrast.^[^ ^29^ ^]^ This behavior suggests that the newly arriving IZO building blocks (as indicated by the enhanced *I_2_*), directly impinging on the Template T1, first nucleate at surface defect sites on the ITO film.^[^ ^31^ ^]^ Moreover, the thickness of this percolation layer is ≈1.9 nm after 11.4 min of the IZO deposition at a sputtering rate of 10 nm h^−1^, which aligns with a 1.6 ± 0.1 nm measurement extracted from the XRR fit result. In growth regime II (11.4–74.6 min), the scattering intensity distribution is predominantly dictated by small domains of structure 2 and the emerging structure 3, which exhibits a spherical form factor with a radius of ≈3 nm. Consistent with the low‐*q_y_* shift observed in Figure 5a, *D_2_* increases, accompanied by reduced ω_2_ from 37 % to 30 %, indicating that the configuration of structure 2 becomes more orderly as the number of arriving building blocks increases. This finding suggests that adsorption‐driven coalescence dominates the growth of structure 2, leading to an effective increase in the thickness of the IZO thin film, represented as peak V1 in Figure 5c.^[^ ^29^, ^32^ ^]^ Notably, no correlated inter‐domain distance is obtained from structure 3 based on the modeling results. However, the H1 peak (marked in Figure 5c), which shifts toward a higher *q_y_* value as an indication of a reduced inter‐domain distance, is attributed to the smallest granular structure 3 appearing at a high *q_y_* position. The intensity *I_3_* rises over sputter deposition time, accompanied by a decrease in the inter‐domain distance of structure 3, resulting in the compactly arranged grain domains shown in the SEM image in Figure 3b. As a result, the increased inter‐domain distance *D_2_* of structure 2 provides space for the accumulation and dense packing of newly formed structure 3. In growth regime III (74.6 min onward), the IZO layer becomes well developed and completely covers the ITO film, as confirmed by XRR measurements, which indicate the formation of one monolayer with a thickness of 11.0 ± 1.0 nm. Subsequently, the growth of structure 2 and structure 3 is primarily driven by the layer growth mode (Frank‐van der Merwe Growth) for the film growth.

**Figure 6:**
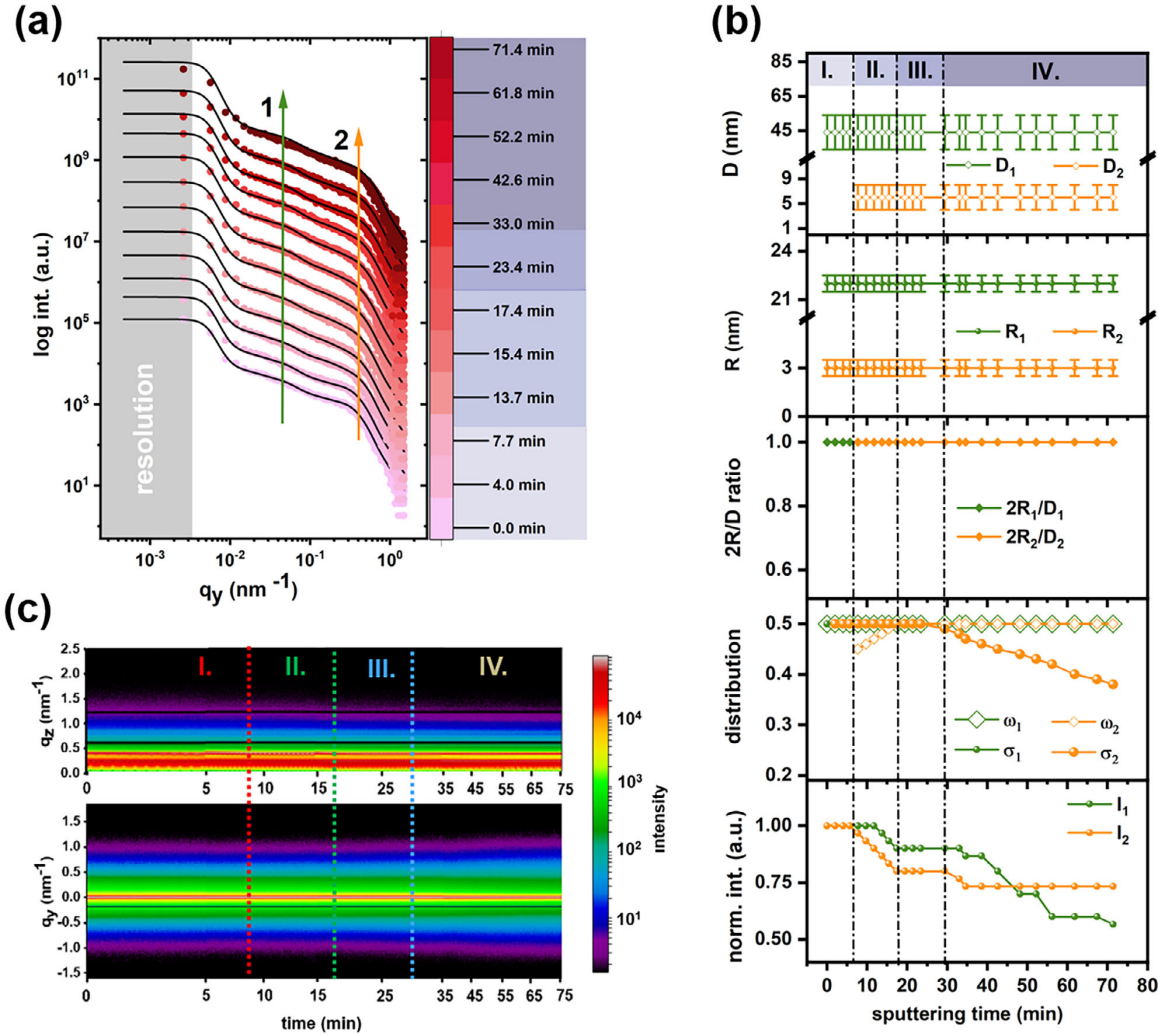
a) Selected horizontal line cuts from the 2D GISAXS data of the Template T2 with increasing sputtering time. The fits are shown as black lines. Curves are shifted along the intensity axis for clarity of the presentation. The marked green arrow and orange arrow show the features of structure 1 and structure 2, respectively. b) Structure‐related parameters extracted from modeling data. The error bars represent the fit parameter tolerance. The 4 observed degradation regimes (I–IV) are indicated. c) 2D mapping of all horizontal and vertical line cuts obtained from the 2D GISAXS data.

For the T2 sample, integrations of selected horizontal line cuts are performed along the Yoneda peak region of ZnO (*q_z_* = 0.587–0.593 nm^−1^). As shown in **Figure** **6**a, the Template T2 exhibits characteristic features (denoted as structures 1 and 2) attributed to the ZnO NP film before IZO sputtering. The absence of any pronounced scattering peaks suggests the high polydispersity of all these related structures. Moreover, changes in peak positions over the sputter time are not visibly predominant. Before the IZO sputtering, two distinct structures with form factors of spheres are modeled for the ZnO NP thin film. As shown in Figure S10b (Supporting Information), structure 1 consists of larger domains with an average radius *R_1_* of 22 nm, a size distribution. σ_1_ of 50%, and a correlated inter‐domain distance (*D_1_*) of 44 nm with a distance distribution ω_1_ of 50%. Structure 2, representing smaller domains, has an average radius *R_2_* of 3 nm with a size distribution σ_2_ of 50%. Compared to the structural evolution on the Template T1, the changes in domain sizes are not observed over the sputter time.

To elucidate the ion bombardment‐induced degradation process during IZO sputtering, the modeling results are quantitatively presented in Figure 6b. According to the diffuse scattering relation $P\left(\vec{q}\right)\propto N{\left|F\left(\vec{q}\right)\right|}^{2}S\left(\vec{q}\right)$,^[^ ^28^ ^]^ where N is the number density of scattering objects, $F\left(\vec{q}\right)$ is the object form factor, and $S\left(\vec{q}\right)$ is the structure factor; the temporal evolution of scattering intensity reflects changes in the number of scattering domains, electron density contrast, and the degree of structural disorder in spatial configuration. Different from the growth dynamics for the Template T1, where the scattering density of small‐domain structures increases over time due to the increasing population of sputtered IZO adatoms, the ZnO film of the Template T2 exhibits a decreasing tendency in scattering intensity for all structures. This behavior implies that no pure IZO layer grows and that physical degradation is the dominant process. Consistent with the XRR and XRD results, this physical degradation ultimately leads to the surface amorphization of the ZnO NP film. To further illustrate the underlying mechanism, the degradation process is divided into four regimes based on the structural evolution characteristics.

In degradation regime I (0–7.7 min), no changes are observed in all relevant structure parameters, which reveals the balance of adsorption and desorption rates of IZO adatoms on the ZnO NP film surface, implying that the surface instability in trapping sufficient adatoms for nucleation is owing to the lack of well‐defined sites on the rough ZnO NP film.^[^ ^33^ ^]^ Moreover, the low deposition rate also allows fewer arriving IZO atoms. In degradation regime II (7.7–17.4 min), the ion bombardment on the ZnO NP surface occurs and sputters away ZnO atoms, as indicated by the diminished scattering intensity in both structures, leading to a loss of well‐defined grain boundaries and reduced populations of small domains. Meanwhile, the correlated inter‐domain distance ω_2_ appears and rises from 45% to 50%, suggesting a more disordered configuration of structure 2 and its rearrangement in spatial configuration. In degradation regime III (17.4–30 min), the process is again driven by the adsorption‐desorption equilibrium, which is similar to what was observed in regime I. This behavior is likely due to the surface roughening caused by the mobilized ZnO atoms during regime II. In degradation regime IV (30 min–71.4 min), ion bombardment continues, resulting in the morphological change on the surface, as evidenced by the reduction of the size distribution σ_2_ from 50% to 38%. Despite these structural modifications, the electron density contrast for small domains remains stable, as reflected in the consistent evolution of *I_2_*. Conversely, the decreasing intensity of *I_1_* is attributed to decreased electron density contrast rather than reduced populations of large domains, suggesting that the high‐energy ions continuously bombard structure 1 and actively interact with surface ZnO atoms. This interaction may involve a complex collisional relaxation process, where energetic ions transfer energy to substrate atoms, causing a cascade of collisions that lead to defects accumulation, disruption of the crystal structure, and eventually amorphization,^[^ ^34^ ^]^ presumably resulting in the formation of voids and vacancies observed in the SEM image (Figure 3d). Moreover, the disordered configurations and loss of electron density contrast are consistent with a lower SLD and a reduced surface roughness due to surface amorphization in XRR results. Specifically, the evolution of the structure factor dictates the ion bombardment‐induced degradation process in degradation regime II, while the form factor predominates in regime IV. Thus, the ultimate surface amorphization of the Template T2 during IZO sputtering primarily results from the ion bombardment‐induced structural changes occurring in the degradation regimes II and IV. As presented in contour plots in Figure 6c, the stable intensity modulations along both *q_z_* and *q_y_* directions indicate the slow degradation process induced by ion bombardment.

The net deposition on Template T1, and physical degradation on Template T2 due to ion bombardment effect can be closely related to the varying arrival rates of IZO species depend on the underlying substrate in terms of ultra‐thin film deposition.^[^ ^35^ ^]^ It is worth noting that the arrival rate of IZO species is given by its relative deposition flux, defined as the ratio between the flux of the incoming IZO neutral atoms and the flux of energetic O^−^ ions. During this experiment, an unusually high ratio of negative O^−^ ion flux to the sputtered IZO atoms is likely present. The target is exposed to air during sample exchange, which facilitates oxidation of the target surface, leading to an increased risk of highly energetic O^−^ ions being formed near the IZO target surface. Therefore, the high flux of energetic O^−^ ions paired with the relatively low deposition rate of IZO atoms leads to the adsorption‐desorption equilibrium in regimes I and III. Additionally, the low‐density, porous ZnO NP film offers weak surface binding compared to the dense ITO film in Template T2, leading to a lower relative deposition flux, allowing a higher probability of physical degradation and adsorption‐adsorption equilibrium.^[^ ^36^ ^]^ Surprisingly, the energy of the arriving particles at the substrate was seemingly still significant despite the high working gas pressure. One explanation could be that the small collision cross section δ(*E*) largely increases the mean free path λ(E) of sputtered particles, indicating high‐energy sputtered particles undergo fewer collisions with gas particles and thus arrive on the ZnO NP film surface with high kinetic energy.^[^ ^37^ ^]^ Therefore, by increasing the process pressure, it is unlikely that the mean free path length of damaging energetic particles is reduced. The corrected energy‐dependent mean free path λ(E) is calculated according to λ(E) = *kT*/(P*δ(*E*)),^[^ ^38^ ^]^ where *k* is Boltzmann constant and δ(*E*) is the energy‐dependent collision cross section according to Somekh.^[^ ^16^ ^]^ To eliminate the ion‐bombardment‐induced damage, a reduced ion flux by pre‐conditioning the target surface to remove excess oxygen or indirect deposition (meaning only scattered IZO particles can reach the substrate), combined with an increased film formation rate, which could be achieved by applying strong magnets to the target, would likely disrupt the adsorption‐desorption equilibrium. Additionally, a growing IZO film would protect the underlying ZnO film from further ion bombardment.

**Figure 7:**
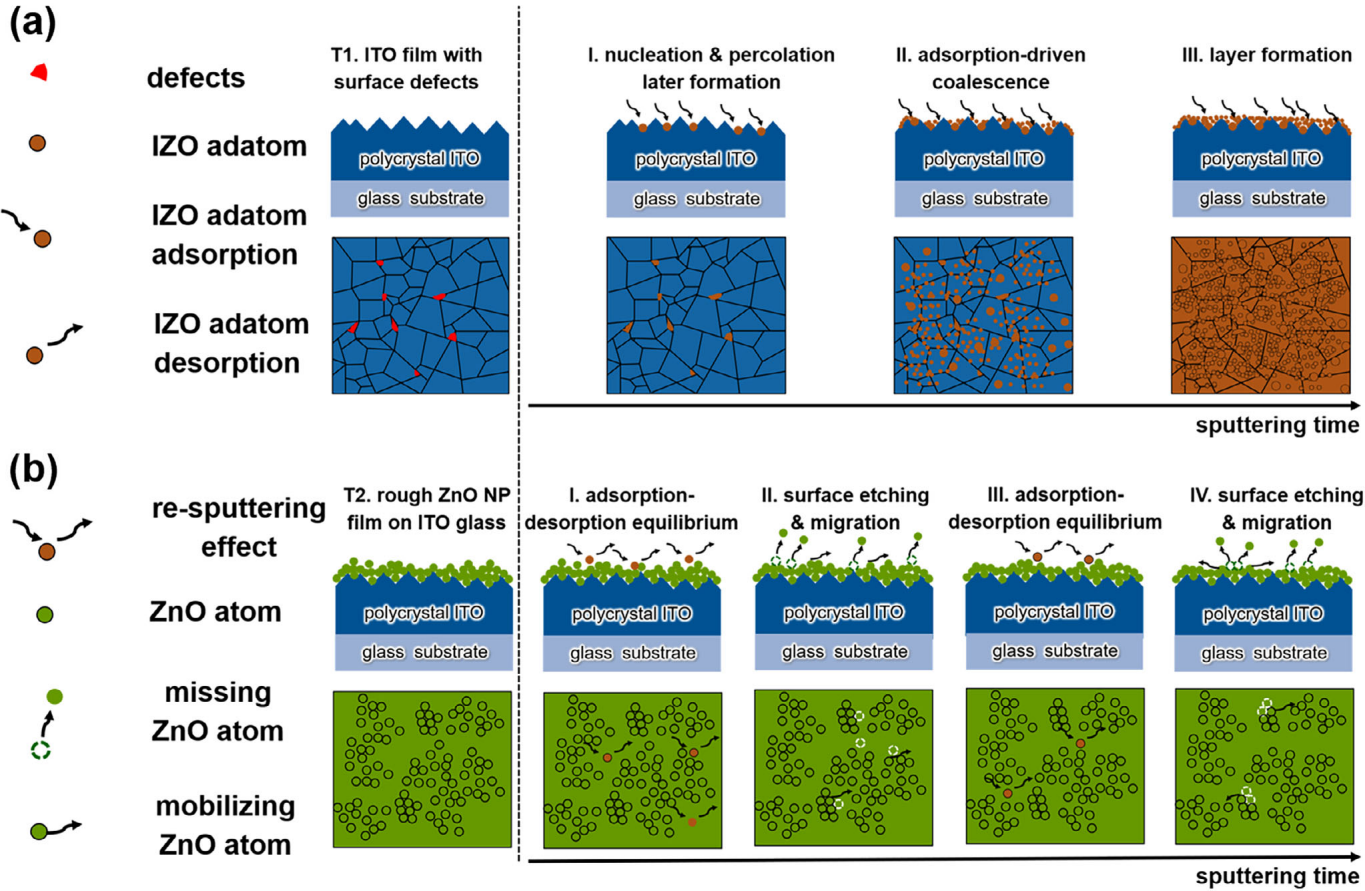
Schematic illustration in side view and top view of a) the film growth regimes of sputter‐deposited IZO on the Template T1 and b) the degradation stages in the case of Template T2, starting with the initial Templates T1 and T2 (very left). The red area, green spheres, and dark orange sphere represent surface defects, ZnO NPs, and IZO domains. The white dashed circles indicate the etched ZnO NPs. Notably, the re‐sputtering effect here refers to the net mass loss, rather than ion‐induced ejection.

To better understand the film growth and ion bombardment‐induced degradation process during IZO sputtering, a visualization of the processes is presented in **Figure** **7** based on the integral analysis results of GISAXS, SEM, and XRR characterizations. As presented in Figure 7a, the Template T1 exhibits surface defects on the commercially sputtered ITO film. Growth regime I is defined by nucleation and percolation formation, where the nucleation occurs at surface defect sites, generating the percolation layer. In growth regime II, vapor‐phase IZO adatoms adsorb onto the ITO surface and coalesce with proximal adatoms, increasing the IZO coverage of the ITO surface and the thickness of the surface layer. In growth regime III, the IZO layer is formed, and the layer growth proceeds. As shown in Figure 7b, the spin‐coated ZnO NP film of the Template T2, which manifests in a porous and rough surface, undergoes four stages in the degradation process. In the degradation stage I, the adsorption‐desorption equilibrium dominates. The rough ZnO nanoparticle surface provides fewer well‐defined adsorption sites, making it less favorable for the stable capture and nucleation of vapor‐phase IZO adatoms. ^[^ ^33^ ^]^ In degradation stage II, the O^−^ ions bombard ZnO NP surface, mobilizing the ZnO atoms by energy transfer and resulting in the physical degradation by sputtering away ZnO atoms. As a result, the spatial configuration of small domains changes, roughening the surface and causing the adsorption‐desorption equilibrium to dominate again in the degradation stage III. In degradation stage IV, the O^−^ bombardment continues and ZnO atom migration dominates, driven by the collisional relaxation process toward thermal equilibrium, resulting in morphological changes such as defect formation and surface smoothing. ^[^ ^39^ ^]^ Therefore, the physical degradation of the Template T2 is characterized by the interplay of the re‐sputtering effect and the collisional relaxation process. The degradation dynamics are comprised of initial adsorption‐desorption equilibrium, physical etching, reestablished adsorption‐desorption equilibrium, and surface amorphization.

### 2.4 Optical and Electrical Properties

**Figure 8:**
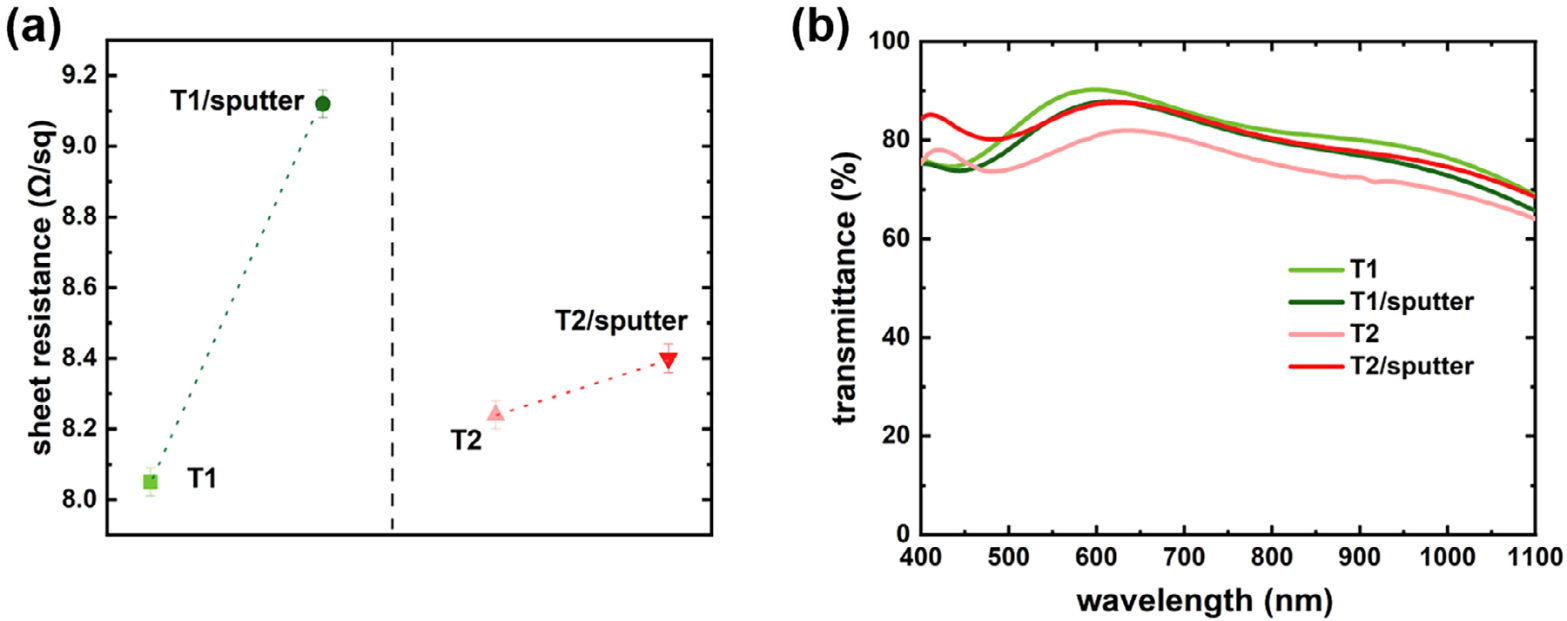
a) Sheet resistance and b) transmittance spectra for the initial Templates T1 and T2, shown together with the final sputtered samples.

To establish the relationship between film morphology and optical and electrical properties, UV–vis‐near infrared spectrophotometry and four‐point probe measurements are performed to evaluate optical transmittance and conductivity. As shown in **Figure** **8**a, the sheet resistance for each sample is determined by averaging multiple measurements. Following IZO deposition, the resistance of the T1/sputter sample increases, which can be attributed to the intrinsically less conductive property of the IZO film compared with the underlying ITO film. Additionally, a higher resistance in the T2/sputter sample than T2 sample can be attributed to the formation of voids and defects at the ZnO surface, as revealed in the SEM images. Regarding optical properties, the optical transmittance is strongly influenced by the film thickness, and in all multilayer samples, it is predominantly determined by the thick ITO layer. The IZO deposition reduces the transmittance of the T1/sputter sample due to the increased overall film thickness and increased surface roughness. A similar reduction is observed for the spin‐coated ZnO NP layer on ITO film in the Template T2 by comparing with the Template T1. In contrast, the smoothing of the ZnO NP film, caused by ion bombardment‐induced surface amorphization, reduces diffuse reflection and scattering to increase transmittance of the T2/sputter sample (Figure 8b).

Based on the above observations, it is inferred that a robust TCO film, such as the ITO film in the Template T1, can still support the subsequent growth of IZO film even after experiencing ion bombardment during sputtering. In contrast, surface amorphization in crystalline films, such as the ZnO NP layer on the Template T2, highlights ion bombardment‐induced structural changes to a susceptible layer. Using a buffering layer, like the ZnO NP film on the ITO film in the Template T2, can alleviate the direct damage of ion bombardment on the underlying TCO film. However, the rough surface and the grain boundaries with associated defects in the ZnO NP buffering layer reduce transmittance and slightly increase sheet resistance, respectively, when comparing the Template T1 and the Template T2. Thus, to mitigate ion bombardment‐induced damage, applying a robust, defect‐free, and uniform buffering layer is favorable for preserving both the conductivity and transmittance of the underlying TCO film. These findings provide important insights for sputtered transparent electrodes in optoelectronic devices, particularly regarding sputter‐induced damage and strategies for its mitigation.

## 3 Conclusion

In conclusion, the dynamics of film growth and ion bombardment‐induced degradation during film deposition via RF magnetron sputtering of IZO have been successfully investigated using in situ GISAXS. It has been demonstrated that energetic particle bombardment, which likely stems from O^−^ ions, can cause physical degradation, including physical degradation and surface amorphization of the spin‐coated ZnO NP film due to its weak surface bonding, despite the high working pressure, whereas an IZO film with a thickness of 12 nm is deposited on the robust, commercially sputtered ITO film. Therefore, it is concluded that increasing the working gas pressure is not an effective method to reduce the energy of highly energetic and damaging particles. SEM images show that the IZO film deposited on the ITO film exhibits well‐defined granular domains with distinct lattice boundaries rather than a smooth, amorphous film, likely due to the lack of energy transfer of sputtered IZO atoms to surface adatoms with moderate kinetic energies of <50 eV. These observations support the theory by Somekh,^[^ ^16^ ^]^ that the collision cross section between sputtered particles and gas particles is energy‐dependent, and therefore thermalization of energetic particles takes place selectively for low‐energy particles. In contrast, the O^−^ ion bombardment‐induced etching leads to the formation of voids and defects on the surface layer of the ZnO NP film. The transmittance spectra and sheet resistance show the impact of morphological changes, caused by the different sputtering mechanisms, on the optoelectronic properties of all the films, providing insights into the behavior of sputtered TCOs for optoelectronic applications. Overall, this experiment highlights the importance and complexity of sputter process conditions for the growth dynamics and that the choice of substrate or the use of buffering layer can impact the ultimate film formation.

## 4 Experimental Section

### Materials

Indium tin oxide (ITO, In_2_O_3_: SnO = 90 wt.%: 10 wt.%) was purchased from Youxuan, Inc. Zinc oxide nanoparticle (ZnO NP) ink (2.5 wt.%, work function ‐4.3eV) was purchased from Sigma‐Aldrich. Amorphous indium zinc oxide target (IZO, In_2_O_3_: ZnO = 90 wt.%: 10 wt.%). Hellmanex III alkaline cleaning concentrate, acetone (purity ≥ 98%), isopropanol (purity ≥ 98%), and ethanol (purity ≥ 98%) were purchased from Carl Roth GmbH & Co. KG.

### Template Preparation for IZO Film Sputtering

The commercial ITO was cleaned by sequentially immersing the substrate in the following solvents in a sonic bath for 15 min: mixed cleaning solution with Hellmanex III: deionized (DI) water (2:98), DI water, acetone, isopropanol, and ethanol. The cleaned ITO glass as Template T1 was ready for the IZO deposition. To prepare Template T2, ZnO NP ink was deposited on a cleaned ITO substrate by spin coating with 3000 rpm for 30 s, followed by the annealing treatment at 120°C for 10 min. The resultant thickness of ZnO is 25 nm ± 5 nm, measured by SEM.

### IZO Film Deposition via RF Sputtering Technique

The chamber pressure for RF sputtering was set to 3.5 × 10^−2^ mbar with pure argon as working gas, and a quartz crystal microbalance (QCM, Inficon) was operated for measuring the IZO sputter deposition rate, which is 10 nm h^−1^. The deposition time for each template was 75 min. All sputter processes were done at room temperature.

### In Situ GISAXS Observation

To probe the dynamic deposition process of IZO on the templates, the sputter deposition system was integrated into a GISAXS setup at the P03/MiNaXS beamline of the PETRA III storage ring at Deutsches Elektronen–Synchrotron (DESY, Hamburg, Germany).^[^ ^40^, ^41^ ^]^ The incident photon energy was 11.83 keV, corresponding to the wavelength of 1.048 Å. The sample‐to‐detector distance (SDD) was set to (3355 ± 2) mm for in situ GISAXS measurement using a Pilatus 1M (Dectris Ltd., Switzerland; pixel size = 172 µm × 172 µm) as the detector. An incident angle of *α_i_* = 0.4° was selected for the in situ measurements, which was above the theoretical critical angles of ITO (0.24°, density 6.8 g cm^−3^), ZnO (0.22°, density 5.8 g cm^−3^), and IZO (0.25°, density 7.2 g cm^−3^). The X‐ray beam was adjusted to a width of 25 µm and a height of 27 µm, thus a large area of ≈0.10 mm^2^ was probed on the samples. To avoid possible X‐ray radiative damage to the sample, we performed repeated movements of the sample in the horizontal direction and checked for beam damage with scans along the sample plane. During the in situ GISAXS experiment, the scattering patterns were continuously recorded at a frame rate of 10 images per second, and motor moves at a rate of $\frac{1}{6}$ mm/s for y‐direction scanning. The GISAXS line cuts were made with the DPDAK software package.^[^ ^42^ ^]^

### Ex situ Characterizations

XRR and XRD measurements were performed on a Bruker D8 ADVANCE X‐ray diffractometer using Cu Kα radiation with a wavelength λ of 1.5418 Å at 40 kV and 40 mA. The reflection patterns were recorded over the 2θ range 0.04°–2.00° (q range with 0.003 Å–0.142 Å) with an increment of 0.002° at the speed of 2 s/step for all samples, and XRR fits were performed with the Motofit tool integrated into Igor Pro software. The refraction patterns were measured over the 2θ range 10°–80° with an increment of 0.002° at a speed of 4 s/step for all samples. SEM measurements were conducted using a Zeiss Gemini NVision 40 with an electron acceleration voltage of 5 kV, a working distance of 3.5 mm, and an aperture size of 10 µm. For all samples, visible‐near‐infrared (Vis–NIR) spectra were measured by a Lambda 35 instrument (PerkinElmer) in normal‐incidence transmission mode with a wavelength range of 400–1100 nm. The scanning speed was set to 240 nm/min with a step size of 1 nm.

## Conflict of Interest

The authors declare no conflict of interest.

## Supporting information



Supporting Information

## Data Availability

The data that support the findings of this study are available from the corresponding author upon reasonable request.
