# Supplementary material for: In Situ GISAXS Study of IZO Deposition via Magnetron Sputtering for Optoelectronic Devices: Film Growth and Ion Bombardment‐Induced Degradation Dynamics
Source: Adv Sci (Weinh). 2025 Nov 7;13(5):e16853. doi: 10.1002/advs.202516853 (PMC12850019; doi:10.1002/advs.202516853)
Supplement: Supplementary file 1 — Supporting Information [file ADVS-13-e16853-s001.pdf]

Supporting Information

In situ GISAXS study of IZO deposition via magnetron sputtering for optoelectronic devices: film growth and ion bombardment-induced degradation dynamics

*Huaying Zhong, Marlene Sophie Härtel, Wei Chen, Lukas V. Spanier, Shanshan Yin, Jiahuan Zhang, Bertwin Bilgrim Otto Seibertz, Bernd Szyszka, Steve Albrecht, Matthias Schwartzkopf, Stephan V. Roth, Peter Müller-Buschbaum**

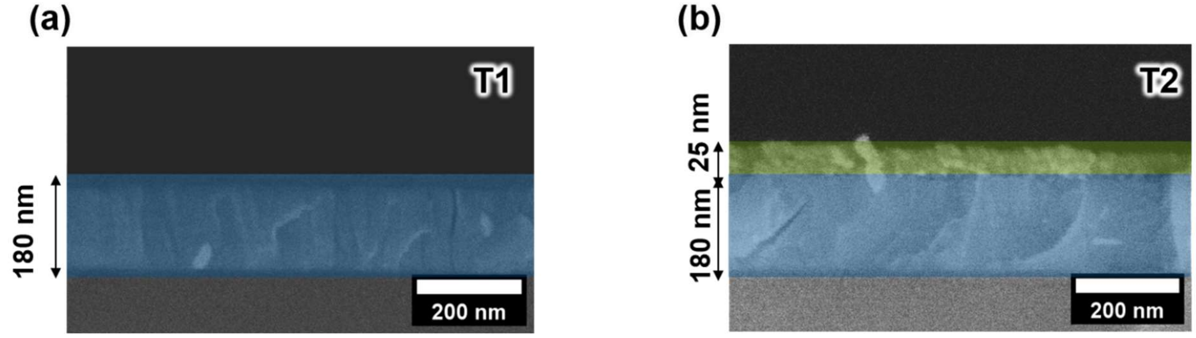

Figure S1. Cross-sectional SEM images of the used templates for IZO sputtering. The ITO film of Template T1 (ITO glass) is approximately 180 ± 20 nm thick, and the spin-coated ZnO NP surface layer of Template T2 is 25 ± 5 nm thick.

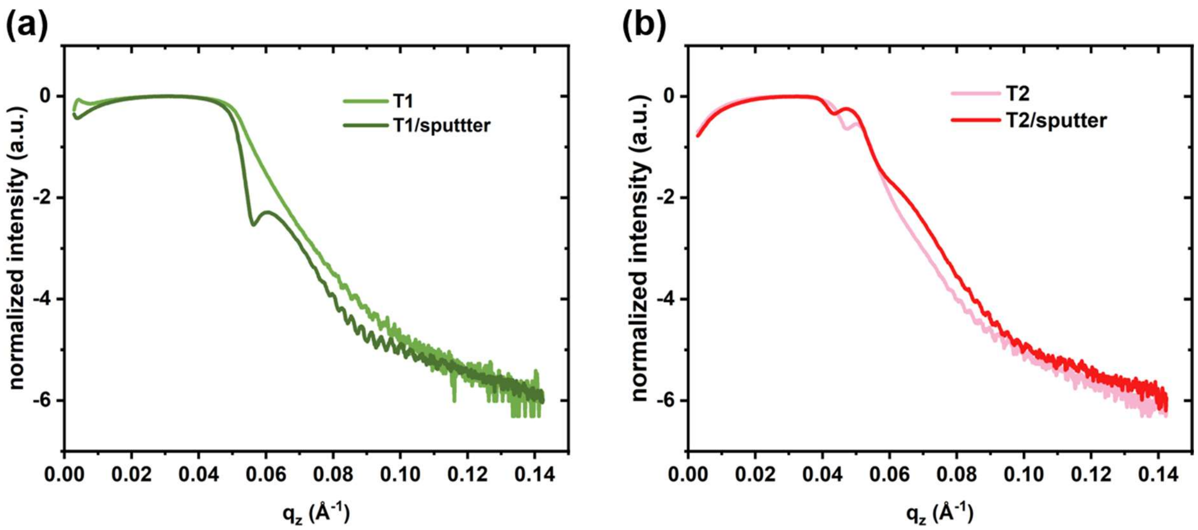

Figure S2. XRR data of samples before and after the IZO sputtering on (a) the Template T1 and (b) the Template T2.

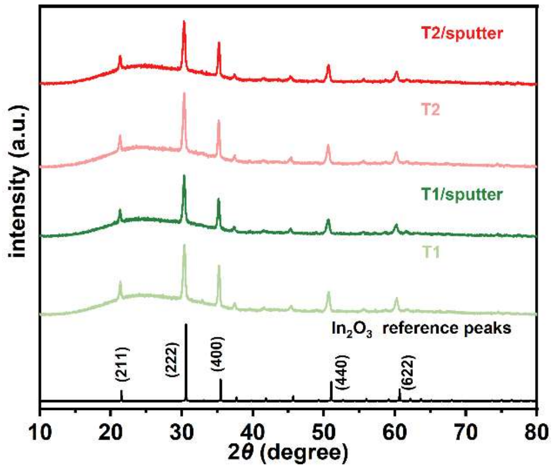

Figure S3. XRD patterns of Template T1 and Template T2 before and after 75 min of IZO sputtering. The In_2O_3 reference peaks are provided from the JCPDS card No. 06-416.

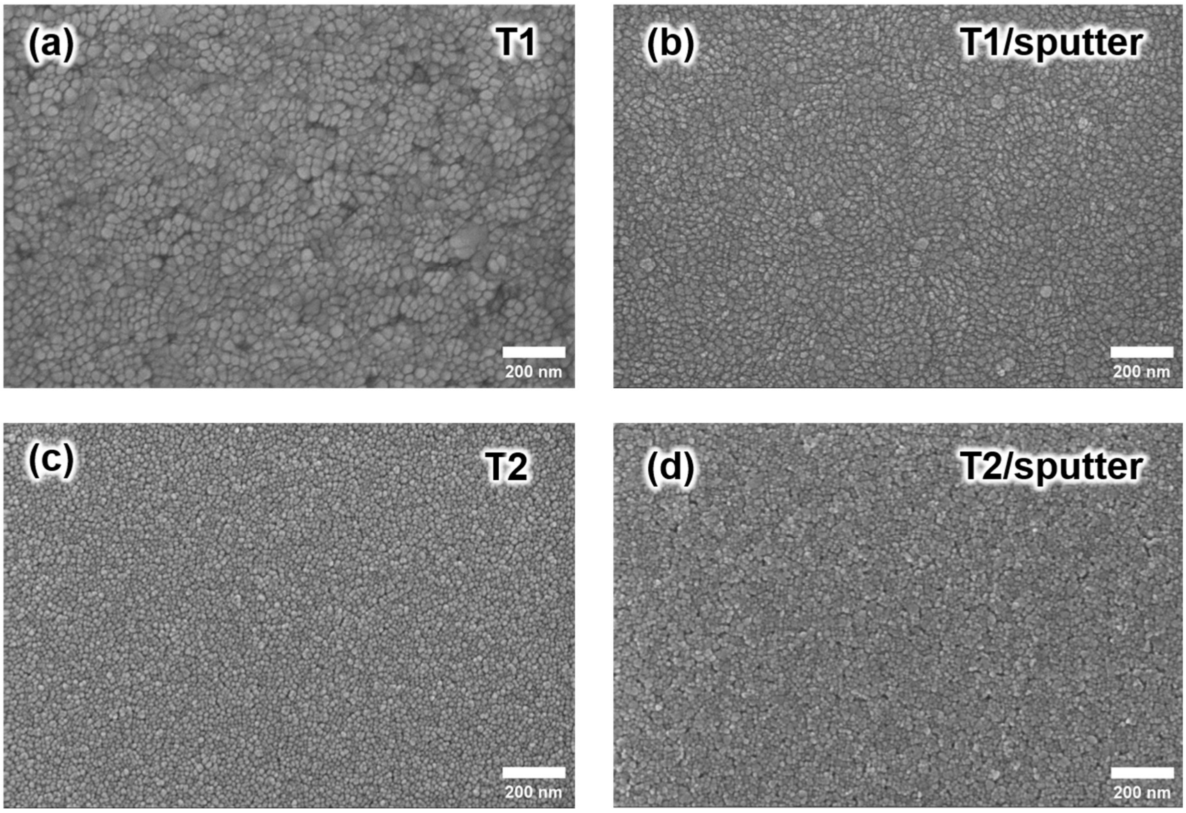

Figure S4. Large area SEM images of Template T1 (a) before and (b) after sputtering, as well as Template T2 (c) before and (d) after sputtering. The defects on the ITO film are visible.

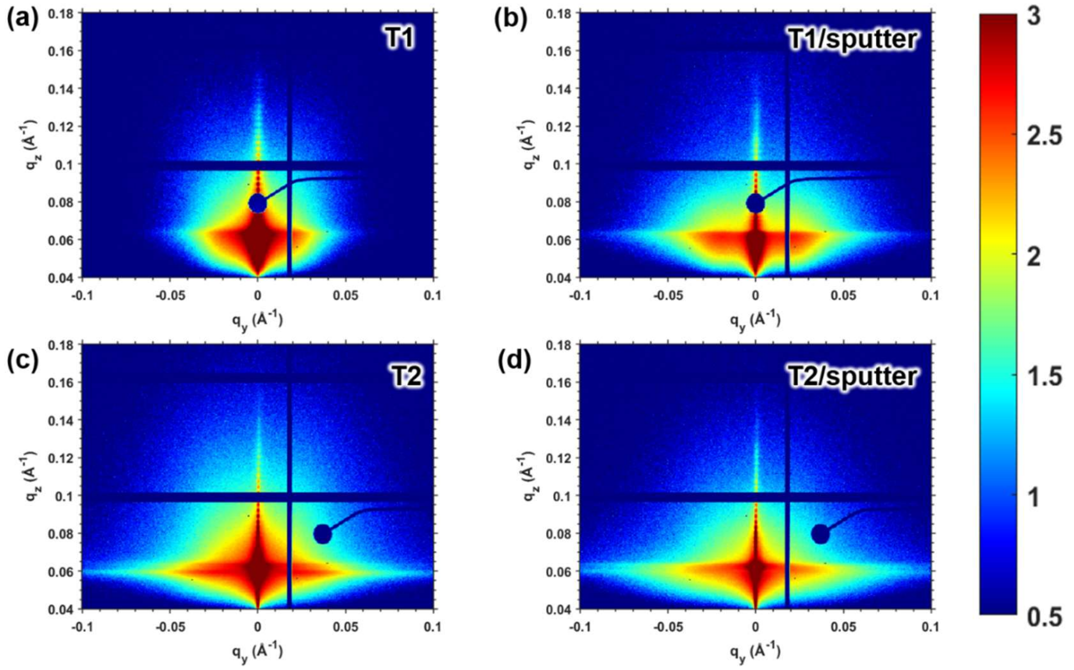

Figure S5. *Ex situ* 2D GISAXS data of Template T1 (a) before and (b) after sputtering, as well as Template T2 (c) before and (d) after sputtering. The beamstop, generally placed at the position of the specular beam to prevent beam damage, is shifted to the right during the measurements of the Template T2, to show the low intensity of the specular beam from the rough surface of the ZnO film of the Template T2.

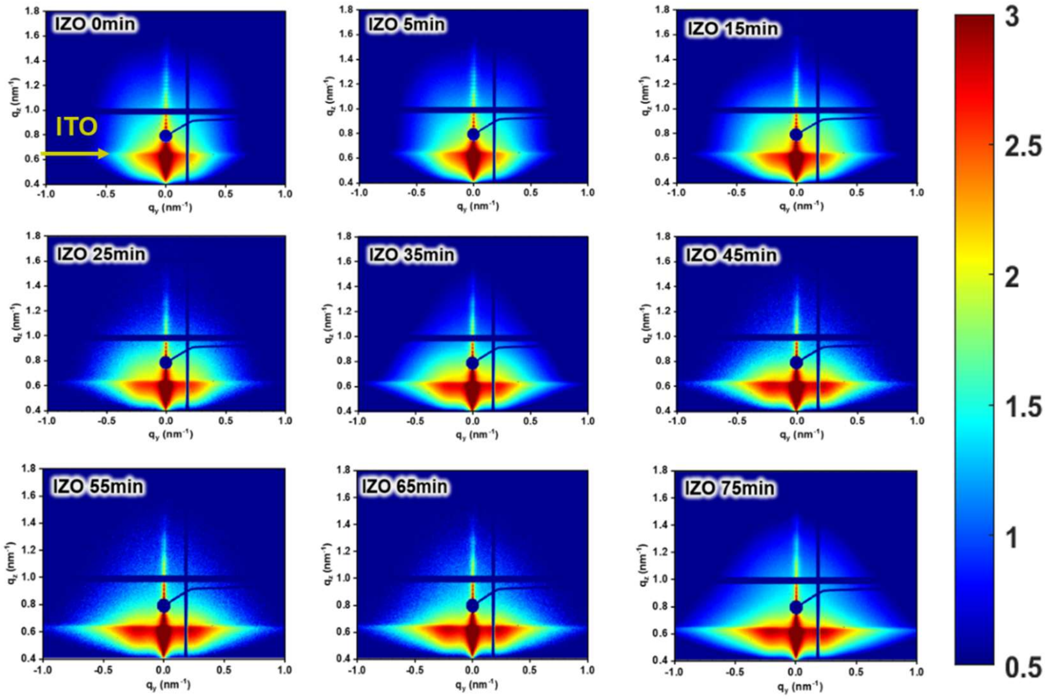

Figure S6. Selected 2D GISAXS data recorded *in situ* during the IZO sputtering on Template T1 over a sputter time of 75 minutes.

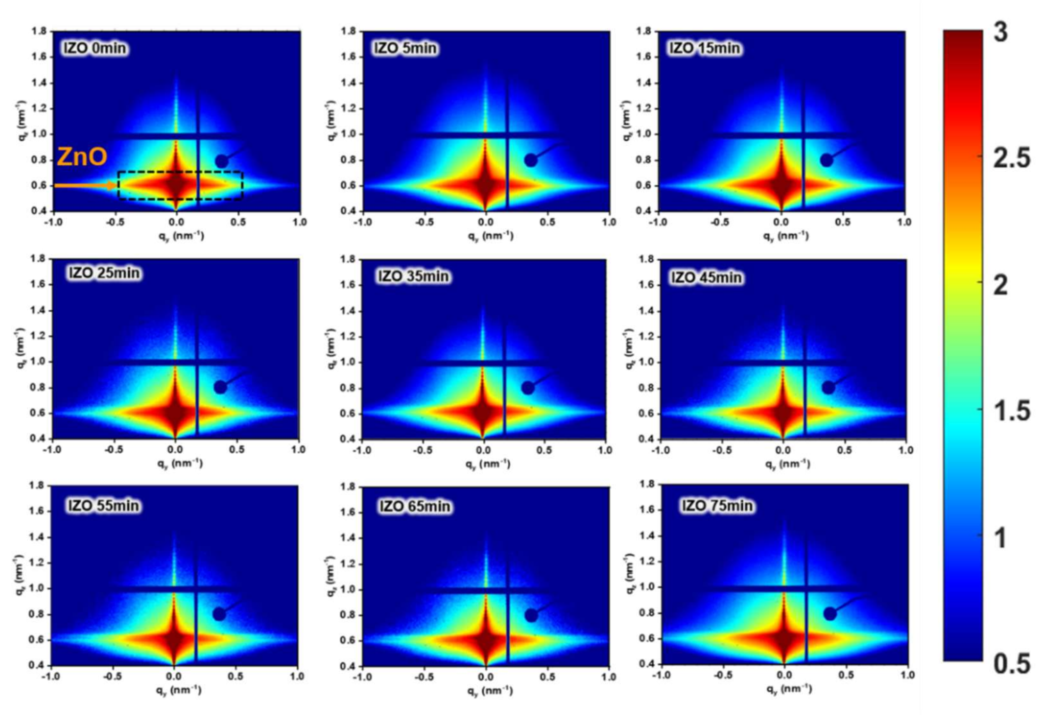

Figure S7. Selected 2D GISAXS data recorded *in situ* during the IZO sputtering on Template T2 over a sputter time of 75 minutes.

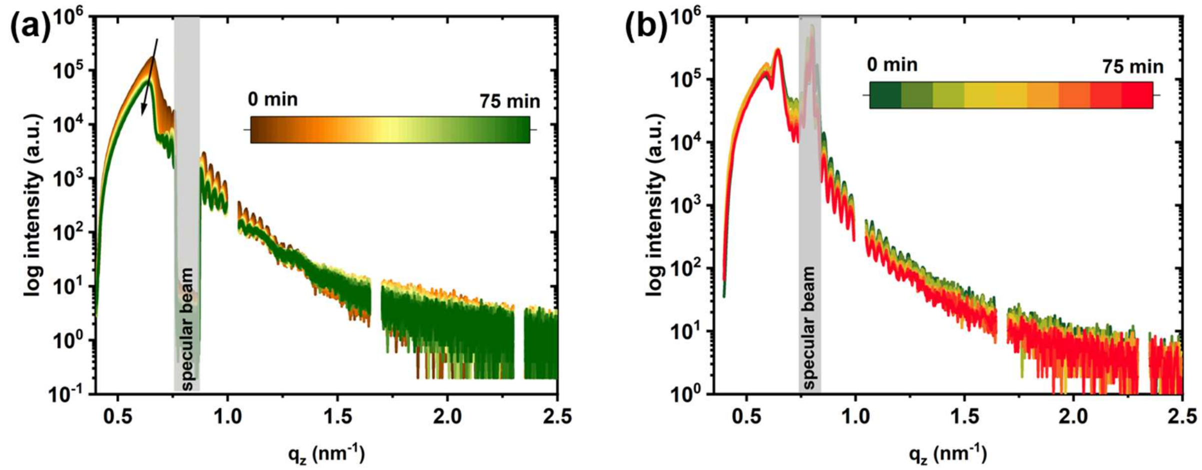

Figure S8. Vertical line cuts of selected 2D GISAXS data on (a) the Template T1 and (b) the Template T2 over 75 minutes of sputter time.

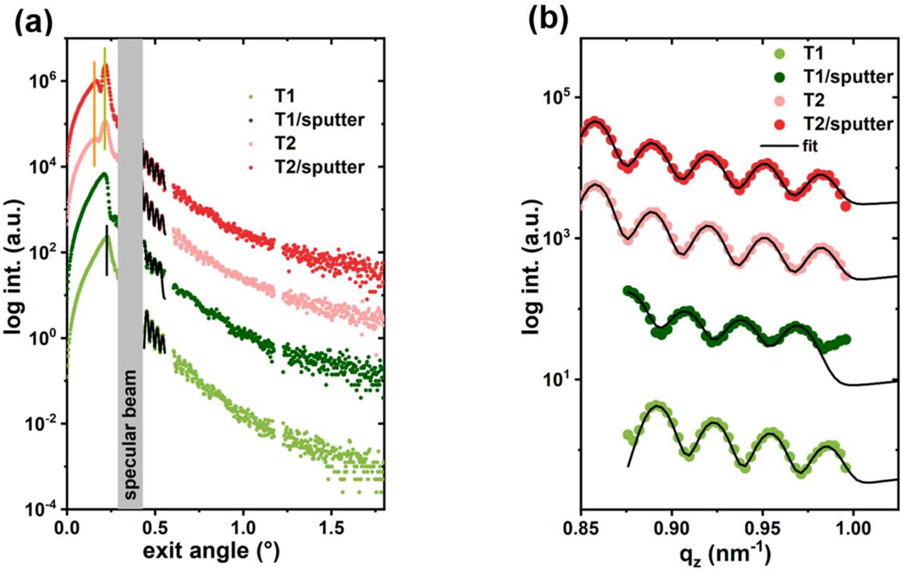

Figure S9. (a) Vertical line cuts of *ex situ* 2D GISAXS data for all measured samples and (b) magnified plots of fitted intensity fringes at the scattering angle in the range of 0.44° to 0.55°.

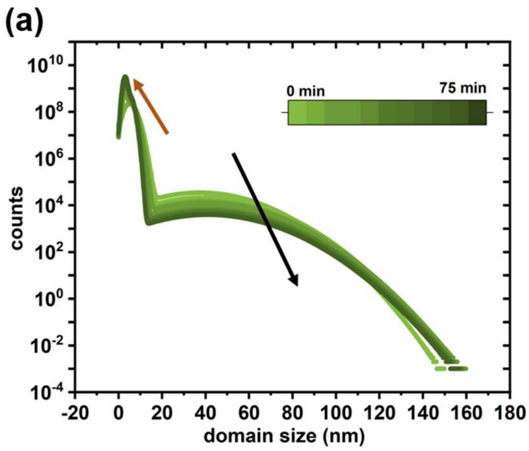

Figure S10. Evolution of the domain size distributions of (a) Template T1 and (b) Template T2 over the sputter time. The black arrow marks the reduced counts of large domains, and the brown arrow indicates the increasing amounts of small domains consisting of structure 2 and structure 3.

XRR data analysis

The analysis of the reflectivity spectra was performed by fits to the experimental data with the reflectivity calculated from Parratt's recursion formula [1]. The XRR reflection patterns were recorded over a 2θ range of 0.04°–2.00°. The specular reflectivity, defined as the ratio of reflected intensity to incident X-ray intensity, was calculated using Fresnel equations and Parratt's recursion formalism, as a function of the perpendicular momentum transfer Q_z, given by

$$Q_z = \frac{4\pi}{\lambda}\sin\theta$$

Here, the wavelength λ of Cu Kα radiation was 1.5418 Å.

All the XRR curves were plotted and fitted as logR vs Q_z without errors. The instrumental resolution (dq/q) was set as 1.95 % for Bruker D8 ADVANCE X-ray diffractometer. The scale factor for the reflectivity measurement was close to 1. The genetic algorithm was applied to do the fit based on the guess range of each parameter.

To start off, the XRR curve of the Template T1 was modeled in a double-layer model with the fitness function χ^2 of 0.012. Based on the initial values of thickness and density of ITO film provided by Youxuan, Inc., a double-layer model consisting of an ITO-substrate interface and ITO bulk layer was applied, apart from a layer of soda-lime glass for the substrate, as well as infinite air at the top of each sample. The obtained reflectivity pattern of the T1/sputter sample

was fitted with a four-layer model including an ITO-substrate interface (interface 1), ITO bulk layer, ITO-IZO interface (interface 2), and IZO layer, and its fitness function χ^2 is 0.010. The fit parameters for the Template T1 before and after the IZO sputtering are presented in **Table S1**.

Table S1. Fit parameters from XRR data of the Template T1 (glass/interface 1/ITO) and the obtained T1/sputter sample after 75 min of IZO sputtering on the Template T1 (glass/interface 1/ITO/interface 2/IZO).

T1 template

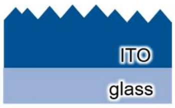

Layers	Thickness (nm)	SLD (10^{-6}Å^{-2})	Roughness (nm)
**IZO**	11.0 ± 1.0	46.8 ± 0.1	3.30 ± 0.05
**interface 2**	1.6 ± 0.1	47.0 ± 0.1	0.80 ± 0.02
ITO	172.2 ± 0.4	55.0 ± 0.3	2.94 ± 0.10
interface 1	4.4 ± 0.4	54.0 ± 2.0	2.00 ± 0.50
glass	-	35.0 ± 3.0	1.49 ± 0.02

T1/sputter

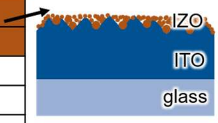

The initial parameters for the Template T2 were based on the modeling result of the Template T1, and a three-layer model was used for the Template T2, consisting of an ITO-substrate interface (interface 1), an ITO bulk layer, and a ZnO nanoparticle layer. The fitness function χ^2 was 0.021. Regarding the T2/sputter sample, the initial intention of adding a single IZO layer on top of the ZnO layer could not fit the reflectivity pattern well. Instead the modification of the ZnO layer was revealed after optimizing the parameters of the top ZnO layer. Thus, the T2/sputter sample was also fitted in a three-layer model with the fitness function χ^2 of 0.041, composed of an ITO-substrate interface (interface 1), ITO bulk layer, and a modified ZnO nanoparticle layer (denoted as ZnO* sputter layer). The fit parameters for the Template T2 before and after the IZO sputtering are presented in **Table S2**.

Table S2. Fit parameters from XRR data of Template T2 (glass/interface 1/ITO/ZnO) and the obtained T2/sputter sample after 75 min of IZO sputtering on the Template T2 (glass/interface 1/ITO/ZnO*sputter).

T2 template

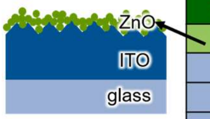

Layers	Thickness (nm)	SLD (10^{-6}Å^{-2})	Roughness (nm)
**ZnO*sputter**	22.2 ± 0.4	27.7 ± 0.3	3.78 ± 0.15
ZnO	21.6 ± 2.0	36.0 ± 0.5	5.00 ± 0.40
ITO	172.2 ± 0.4	55.5 ± 0.3	2.95 ± 0.16
interface 1	4.4 ± 0.4	54.0 ± 4.0	2.00 ± 0.80
glass	-	28 ± 3.0	1.51 ± 0.05

T2/sputter

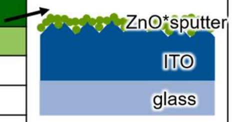

Additionally, by comparing with the value of reduced chi-squared for each curve, a smaller χ^2 indicates a better agreement between the fitted and experimental XRR data, as presented in **Figure S11.**

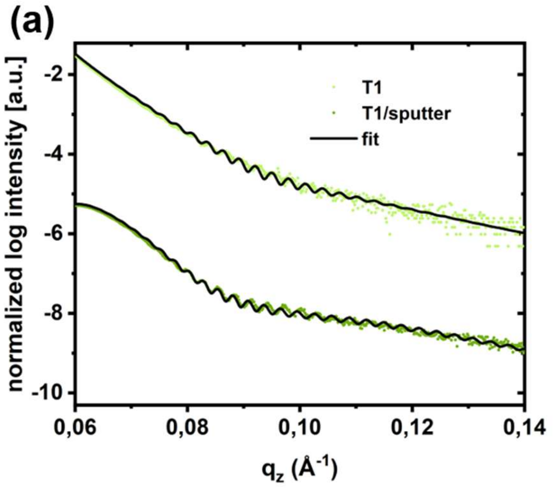

Figure S11. The zoom-in plots of XRR curves for (a) the Template T1 and the Template T2 before and after the IZO sputtering.

GISAXS data analysis

The scattering plane is defined by the incident and exit angles α_i and α_f , and scattering outside this plane is probed under an out-of-plane angle φ.[2] The scattering wave vector $\boldsymbol{q}$ in GISAXS geometry is defined by

$$\boldsymbol{q}=\begin{pmatrix} q_x \\ q_y \\ q_z \end{pmatrix}=\frac{2\pi}{\lambda}\begin{pmatrix} \cos(\alpha_f)\cos(\varphi)-\cos(\alpha_i) \\ \cos(\alpha_f)\sin(\varphi) \\ \sin(\alpha_f)+\sin(\alpha_i) \end{pmatrix}$$

where α_i is the incident angle, α_f and φ are the exit angles of in-plane and out-of-plane directions, respectively.

Extract SLD via vertical line cut

In the region of total external reflection, α_i and α_f are so small that the influence of the periodic crystal structure can be neglected, and a description based on one mean refractive index is appropriate.[24] The phase velocity in a material is larger than in vacuum, the refractive index n of the medium is slightly smaller than 1, and can be written as

$$n = 1-\delta + i\beta$$

where δ is the dispersion term, and the imaginary part β is the absorption term. The critical angle of total external reflection of a material is given by

$$\alpha_c \approx \sqrt{2\delta}$$

The critical value of momentum transfer q_z is q_c , which is related to SLD and given by [3]

$$q_c = \frac{4\pi \sin \alpha_c}{\lambda} = \sqrt{16\pi SLD}$$

Therefore, the following information can be extracted based on the Yoneda peaks of ITO film and ZnO NP film.

	ITO film	IZO film	ZnO film
q_c (nm^{-1})	0.66 ± 0.01	0.63 ± 0.01	0.58 ± 0.01
Critical angle α_c	0.23 ± 0.01°	0.20 ± 0.01°	0.16 ± 0.01°
SLD	54.8 ± 0.2	46.6 ± 0.2	27.0 ± 0.3

Characteristic structures of structures via modelling of horizontal line cuts

To simplify the GISAXS data analysis, the local monodisperse approximation (LMA) in the framework of the distorted-wave Born approximation (DWBA) is applied to model the horizontal line cut data. The probability of a scattering event is determined by the total differential scattering cross-section. The diffuse scattering from a surface is then given by

$$\frac{d\sigma}{d\Omega} = \frac{A\pi^2}{\lambda^2} |\Delta|^2 |T_i|^2 \left|T_f\right|^2 P(\vec{q})$$

Where A denotes the illuminated area by X-ray, $|\Delta|^2$ is the scattering contrast function, T_i and T_f are the Fresnel transimission coefficients of the incident and scattered beam, respectively. $P(\vec{q})$ is the diffuse scattering factor and given by

$$P(\vec{q}) \propto N|F(\vec{q})|^2 S(\vec{q})$$

This scattering factor holds for the monodisperse objects with the number density of scattering objects N, the object form factor $F(\vec{q})$, and the structure factor $S(\vec{q})$. The form factor $F(\vec{q})$

accounts for the shape, size, and orientation of the scattering objects, while the structure factor $S(\vec{q})$ involves spatial distribution, mean distance, and distance fluctuation of the objects.

Here, two different shapes are used as the form factors. The respective form factors are

$$F_s(\vec{q}) = 4\pi R^3 \frac{\sin(qR) - qcos(qR)}{(qR)^3} exp[iq_z R]$$

for ZnO spheres with radius R, and

$$F_c(\vec{q}) = 2\pi H R^2 \frac{J_1(q_r R)}{q_r R} sin\left[(q_r \frac{H}{2}\right] exp\left[iq_z \frac{H}{2}\right]$$

for ITO cylinders with radius R and height H.

The structure factor $S'(\vec{q})$ is related to the mean interdomain distance D and given by

$$S'(\vec{q}) = -\frac{1 - \phi^2(\vec{q})}{1 + \phi^2(\vec{q}) - 2\phi(\vec{q})\cos\left(|\vec{q}|D_c\right)}$$

Where $\phi(\vec{q}) = exp[\pi\omega_D^2 D_c^2 |\vec{q}|^2]$ with ω_D being the standard deviation of a Gaussian distribution at D_c. Since the horizontal cuts are performed at a specific q_c position (Yoneda peak) with a limited pixel area, the q_z is constant and approximated as $q_z \approx 0$. Therefore, the radii and the correlated inter-domain distance of different structures can be resolved by modelling. Notably, the low q area is dominated by the resolution function.

References

[1] L. G. Parratt, *Phys. Rev.* **1954**, *95*, 359.

[2] G. Benecke, W. Wagermaier, C. Li, M. Schwartzkopf, G. Flucke, R. Hoerth et al., *J. Appl. Crystallogr.* **2014**, *47*, 1797.

[3] M. Nayak, G. S. Lodha, *J. At. Mol. Opt. Phys.* **2011**, *2011*, 649153.

[4] P. Müller-Buschbaum, in *Applications of Synchrotron Light to Scattering and Diffraction in Materials and Life Sciences*, Springer, Berlin, **2009**, pp. 61-89.
